# Two modes of transvection at the *eyes absent* gene of *Drosophila* demonstrate plasticity in transcriptional regulatory interactions in *cis* and in *trans*

**DOI:** 10.1371/journal.pgen.1008152

**Published:** 2019-05-10

**Authors:** Katherine Tian, Rachel E. Henderson, Reyna Parker, Alexia Brown, Justine E. Johnson, Jack R. Bateman

**Affiliations:** Biology Department, Bowdoin College, Brunswick, Maine, United States of America; Geisel School of Medicine at Dartmouth, UNITED STATES

## Abstract

For many genes, proper gene expression requires coordinated and dynamic interactions between multiple regulatory elements, each of which can either promote or silence transcription. In *Drosophila*, the complexity of the regulatory landscape is further complicated by the tight physical pairing of homologous chromosomes, which can permit regulatory elements to interact in *trans*, a phenomenon known as transvection. To better understand how gene expression can be programmed through *cis-* and *trans-*regulatory interactions, we analyzed transvection effects for a collection of alleles of the *eyes absent* (*eya*) gene. We find that *trans*-activation of a promoter by the *eya* eye-specific enhancers is broadly supported in many allelic backgrounds, and that the availability of an enhancer to act in *trans* can be predicted based on the molecular lesion of an *eya* allele. Furthermore, by manipulating promoter availability in *cis* and in *trans*, we demonstrate that the eye-specific enhancers of *eya* show plasticity in their promoter preference between two different transcriptional start sites, which depends on promoter competition between the two potential targets. Finally, we show that certain alleles of *eya* demonstrate pairing-sensitive silencing resulting from *trans*-interactions between Polycomb Response Elements (PREs), and genetic and genomic data support a general role for PcG proteins in mediating transcriptional silencing at *eya*. Overall, our data highlight how *eya* gene regulation relies upon a complex but plastic interplay between multiple enhancers, promoters, and PREs.

## Author summary

Gene regulation requires interactions between regions of DNA known as regulatory elements, which, in combination, determine where and when a gene will be active or silenced. Some genes use just a few regulatory elements, whereas others rely on highly complex interactions between many different elements that are poorly understood. While we typically imagine regulatory elements interacting with one another along the length of a single chromosome, in a curious phenomenon called transvection, elements can communicate between two different chromosomes that are held in close proximity. Here, we use the study of transvection to better understand how different regulatory elements contribute to the expression of *eyes absent* (*eya*), a gene required for proper eye development in *Drosophila*. Our data show that a class of elements that initiate *eya* gene expression, called promoters, will compete with one another for activation by *eya*’s enhancers, a second class of regulatory element, with the promoter that is closest to the enhancers being the favored target for activation. Furthermore, our study of transvection uncovers an important role for a silencing element, called a PRE, in opposing *eya* gene expression. Overall, our study sheds new light on how different elements combine to produce patterned expression of *eya*.

## Introduction

The eukaryotic genome is rich in regulatory elements whose combined inputs lead to proper execution of programmed patterns of gene expression. Regulatory elements that promote gene expression include promoters, where RNA polymerases begin transcription of genes, and enhancers, which bind to transcriptional activator proteins and are thought to physically interact with promoters via looping, thereby recruiting or activating RNA polymerases [1, 2]. Conversely, other DNA elements play roles in preventing transcription locally, including Polycomb Response Elements (PREs), which bind to complexes of proteins known as the Polycomb Group (PcG) and can ultimately create a silenced chromatin domain via the histone mark H3K27^me3^ [3, 4]. While our ability to identify these types of regulatory elements has grown with increasing accuracy via the refinement of sophisticated genomic approaches, our understanding of how specific elements interact with one another across diverse tissues remains incomplete.

**Fig 1:**
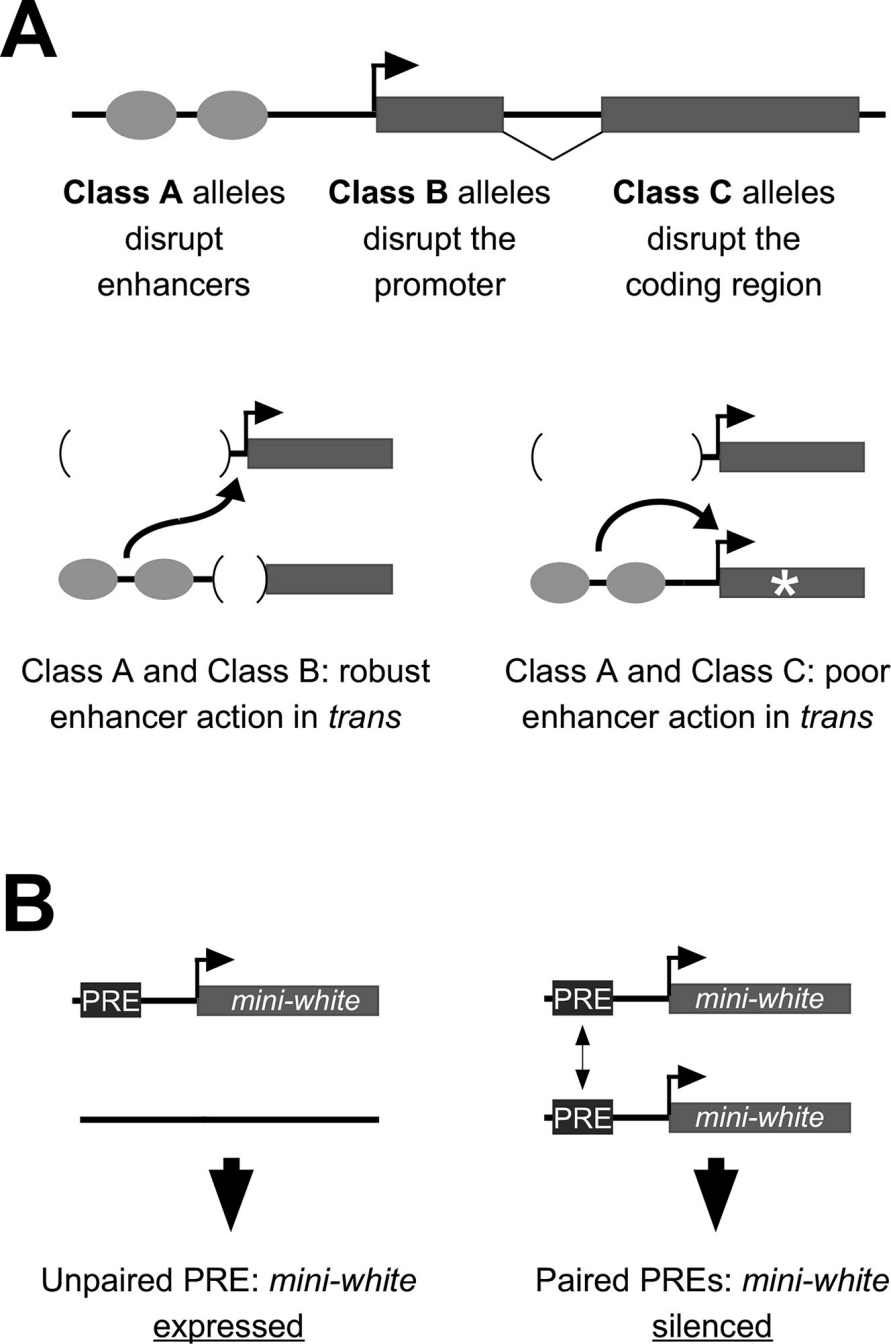
Models for transvection via enhancer action in *trans* and pairing-sensitive silencing. **A**, mutant alleles of *yellow* can be placed into at least three classes based on their molecular lesions: Class A includes deletions of enhancers and insertions of insulator elements between the enhancers and the promoter; Class B includes point mutations and deletions within the core promoter and transposon insertions in either the promoter or the 5’ UTR; Class C includes point mutations and indels in the coding region. Class A and Class B alleles complement when paired, indicating robust enhancer action in *trans*, whereas complementation between Class A and Class C is either non-existent or too weak to be detected, indicating poor enhancer action in *trans*. **B**, a PRE fused to a *mini-white* transgene typically permits expression in a hemizygous transgenic insertion, but silences expression when insertions are homozygous, reflecting pairing-sensitive silencing.

In *Drosophila*, specificity of interactions between regulatory sequences is further complicated by the phenomenon of somatic homolog pairing, where homologous chromosomes are held in close proximity in virtually all somatic cells of the organism [5]. A growing body of data supports that somatic homolog pairing permits regulatory elements on one homolog to interact with those on the homologous chromosome, a phenomenon coined transvection by its discoverer, Ed Lewis [6]. The term transvection encompasses several types of pairing-dependent genetic interactions, including those that positively impact gene expression, as is the case when an enhancer on one chromosome acts in *trans* to activate transcription from a promoter on the homologous chromosome, or that negatively impact gene expression, as observed in some cases when PREs interact in *trans*, which is thought to increase the efficacy of PcG proteins bound to the PRE in silencing transcription (Fig 1) [5, 7].

The study of transvection typically relies on specific mutant backgrounds and/or transgenic organisms with defined constructs placed at equivalent positions on homologous chromosomes. For example, enhancer action in *trans* has been studied extensively for the *yellow* gene of *Drosophila*, which is required for pigmentation of the adult cuticle [8, 9]. The *yellow* gene has a simple structure, with a single promoter and transcription start site (TSS) and several well-defined tissue-specific enhancers, and is rich in classical alleles that impact gene expression. Notably, enhancer action in *trans* at *yellow* appears tightly regulated; intragenic complementation via transvection is observed only between two types of alleles, those in which enhancers are deleted or otherwise prevented from interacting with the *yellow* promoter in *cis* (“Class A” alleles), and those in which the promoter region is compromised by deletion, mutation, or nearby transposon insertion (“Class B” alleles) [9] (Fig 1A). In contrast, alleles of *yellow* that have an intact promoter but carry mutations in the coding region of the gene, known as “Class C” alleles, fail to complement Class A alleles despite carrying functional enhancers that would otherwise be available to act in *trans* (Fig 1A). The failure of the *yellow* enhancers of Class C alleles to act in *trans* has been interpreted to be due to their preference for a promoter in *cis*; it is only when the *cis*-promoter is somehow compromised, as in Class B alleles, that the yellow enhancers are released to act in *trans*, suggesting a hierarchical regulation of potential promoter targets for the yellow enhancers.

Several other examples of enhancer action in *trans* show evidence that enhancers prefer to act on a promoter in *cis* relative to a promoter in *trans* [10–14]. However, in these cases, activation of a *trans*-promoter is attenuated, but not eliminated, in the presence of a promoter in *cis*. A simple interpretation is that *cis*-preference is a global phenomenon that is relevant to many enhancers in *Drosophila*, and that *yellow* represents an extreme case where *cis*-preference is strong enough to reduce *trans*-activation to undetectable levels. However, aside from analyses at *yellow*, there has yet to be further characterization of transvection in *Drosophila* that uses a diverse collection of alleles analogous to the Class A, Class B, and Class C alleles of *yellow*.

The *eyes absent* (*eya*) gene encodes an evolutionarily conserved transcriptional co-activator with protein phosphatase activity that is a component of the Retinal Determination Network (RDN) of transcriptional regulators required for normal eye development in *Drosophila* [15, 16]. Eye-specific loss of *eya* function can cause an “eyeless” phenotype in adult flies, whereas ectopic expression of *eya* can lead to development of eye tissue elsewhere in the body [16, 17]. Eya functions in part via the formation of a complex with the DNA-binding Sine oculis (So) protein, thereby acting as a bipartite transcription factor that regulates RDN gene expression and coordinates other downstream processes of eye differentiation [18].

**Fig 2:**
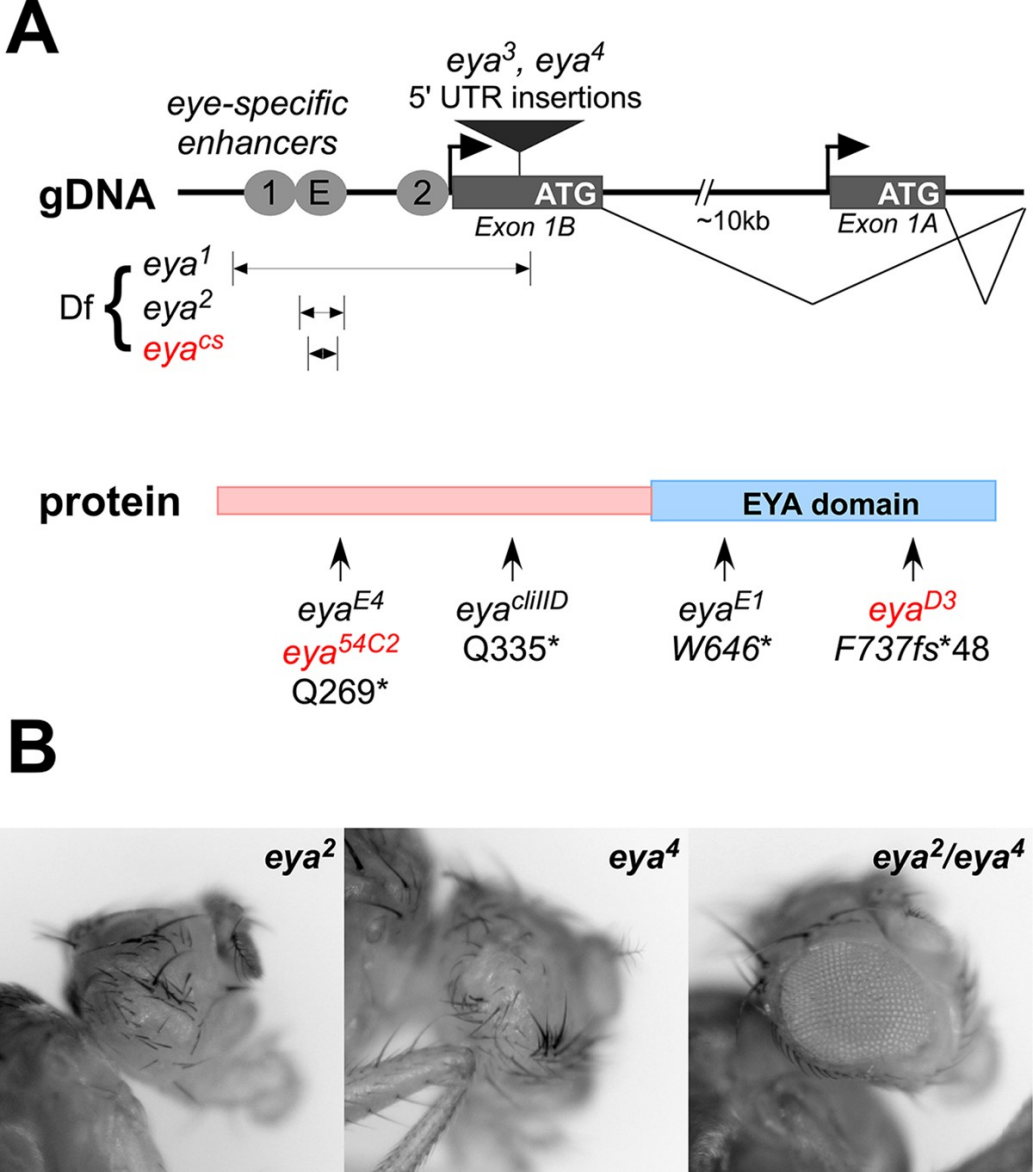
Classical alleles of *eya*. **A**, top, alleles affecting regulatory sequences mapped to a schematic of the locus. Transcription of *eya* initiates from two major promoters, *eya-B* and *eya-A*, which are separated by approximately 10 kb. Several eye-specific enhancers are located upstream of the *eya-B* promoter; E is active in early instar discs and is required for eye development, whereas 1 (also known as IAM) and 2 drive expression in later disc tissues [24–26]. Other fragments identified as candidate late enhancers in other regions of the locus are omitted for simplicity. Alleles *eya*^*1*^, *eya*^*2*^, and *eya*^*cs*^ delete eye-specific enhancer sequences, and *eya*^*3*^ and *eya*^*4*^ carry retrotransposon insertions into the 5’ UTR of exon 1B. Bottom, alleles with point mutations or indels mapped to the Eya protein structure. Alleles in red had their molecular lesions characterized in this work (see S1 File). The *eya*^*D3*^ allele carries an indel in exon 5 where a TT doublet is substituted by CCCCCCCCCG, creating a frameshift at F737 and resulting in a C-terminus of 48 random amino acids in place of the usual 23. **B**, example of transvection between *eya*^*2*^ (Class A) and *eya*^*4*^ (Class B), first demonstrated by Leiserson et al. [27]. Homozygosity of either allele results in eyeless flies, but *trans*-heterozygotes produce an eye of approximately ¾ size via enhancer action in *trans*.

The *eya* gene structure contains two major transcriptional start sites, with the first exon of the *eya-B* (also known as Type I) transcript encoded roughly 10kb upstream of that of *eya-A* (Type II) (Fig 2A). Each of the alternate first exons splices to common second through fifth exons that together encode the majority of the protein. Presumed null mutations in the coding region result in embryonic lethality due to the requirement of *eya* activity in diverse tissues at early stages of development, likely via transcription from the *eya-A* promoter [19–23]. In contrast, retrotransposon insertions into Exon 1B result in homozygous viable flies entirely lacking compound eyes and ocelli, suggesting that the transcript initiated from the *eya-B* promoter is required for development of eye structures [24]. Upstream of the *eya-B* promoter is approximately 9 kb of non-coding sequence that carries several enhancers for distinct eye tissues [24–26], and a DNA fragment carrying this 9 kb fragment can fully recapitulate the wild-type *eya* expression pattern in third instar larval eye discs [25].

Transvection has been previously demonstrated for alleles of *eya* that are analogous to the Class A and Class B alleles of *yellow* [27]. Specifically, *trans*-heterozygous flies carrying *eya*^*2*^, an enhancer deletion, and *eya*^*4*^, an insertion in the 5’ UTR of Exon 1B, result in pairing-dependent rescue of eye development, likely via enhancer action in *trans*. Furthermore, several other alleles of *eya* are structurally similar to Class C alleles of *yellow*, with missense and nonsense mutations in the *eya* coding region, and several show some degree of complementation with *eya* Class A alleles that could be consistent with transvection [19, 20].

Here we demonstrate that Class A alleles of *eya* complement all Class B and Class C alleles tested, demonstrating that enhancer action in *trans* is less strictly regulated at *eya* relative to *yellow*. However, complementation by Class B is consistently stronger than that by Class C across several genetic backgrounds, suggesting that the availability of enhancers to act in *trans* differs depending on the molecular lesion in *eya*. Furthermore, by manipulating promoter availability in *cis* and in *trans*, we show that the eye-specific enhancers of *eya* show preference for the *eya-B* promoter relative to the *eya-A* promoter, and that the preference depends on competition between the two promoter targets. Finally, genetic interactions between *eya* Class A alleles demonstrate pairing-sensitive silencing, and genetic and genomic data support a role for PcG proteins in mediating *eya* transcriptional silencing.

## Results

To date, few loci have been studied in detail for transvection effects using a large number of classical mutations. Transvection via enhancer action in *trans* was previously demonstrated at the *eya* locus, but only one allelic combination was reported in detail (Fig 2B) [27], whereas many more alleles are described in the literature. Thus, to better understand how regulatory regions can communicate in *cis* and in *trans*, we quantitatively characterized transvection effects of a collection of *eya* alleles.

To begin, we gathered *Drosophila* stocks carrying known *eya* alleles and classified them based on their molecular lesions. For those alleles lacking molecular characterization, we identified lesions by sequencing genomic DNA and other methods (Fig 2B; see S1 File). Following the classification scheme of Morris et al. [9], we categorized three alleles, *eya*^*1*^, *eya*^*2*^, and *eya*^*cs*^, as Class A alleles, with each carrying a deletion of enhancer sequences upstream of the eye-specific *eya-B* promoter [16, 24, 26]. In contrast, the Class B alleles *eya*^*3*^ and *eya*^*4*^ carry transposon insertions in the 5’ UTR near the *eya-B* promoter [24], and the Class C alleles *eya*^*E1*^, *eya*^*E4*^, *eya*^*cliIID*^, *eya*^*54C2*^, and *eya*^*D3*^ carry point mutations and/or indels in the *eya* coding region that is common to *eya-A* and *eya-B* isoforms [16, 22, 28]. Note that Class C alleles of *eya* are embryonic lethal as homozygotes or as *trans*-heterozygotes with other Class C alleles, reflecting the requirement for *eya* function during embryogenesis [19, 20], whereas Class A and Class B alleles are homozygous viable, suggesting that they primarily impact eye development.

**Fig 3:**
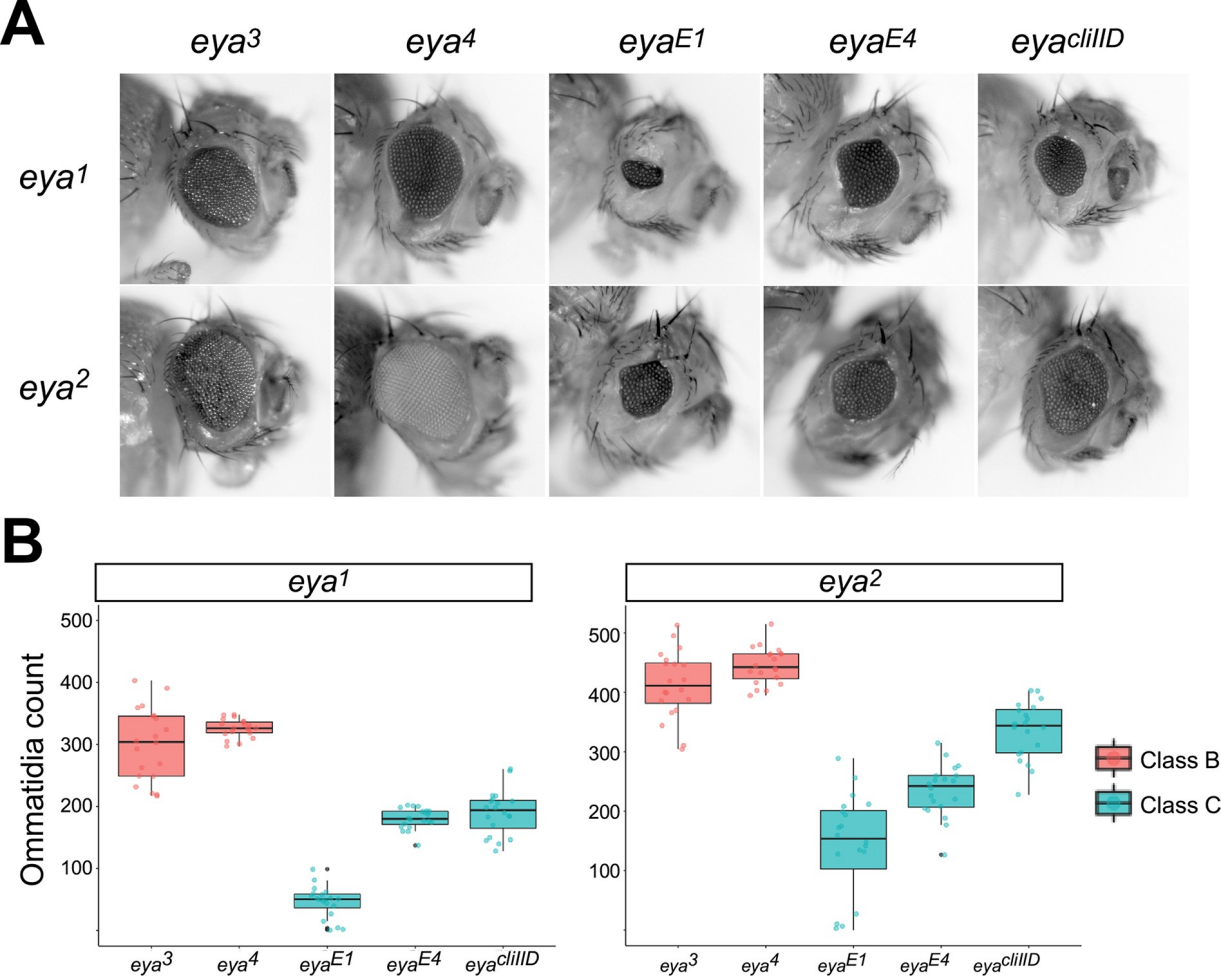
Transvection via enhancer action in *trans* is supported by diverse alleles of *eya*. **A**, eyes of representative flies carrying the Class A allele indicated at left and the Class B or Class C allele indicated above. **B**, quantification of eye development for flies carrying either *eya*^*1*^ (left graph) or *eya*^*2*^ (right graph) and the Class B or Class C alleles indicated below. For each genotype, approximately 20 eyes from 10 flies were scored for the number of ommatidia.

To assess enhancer action in *trans*, we created flies carrying a Class A allele on one homolog and either a Class B allele or a Class C allele on the other homolog (Fig 3). We initially focused on the strong Class A alleles *eya*^*1*^ and *eya*^*2*^, each of which has a completely eyeless phenotype as a homozygote [16, 27]. To quantify the strength of transvection in *trans*-heterozygous flies, we scored the number of ommatidia in adult fly eyes from each genotypic combination (see Materials and Methods). All seven Class B and Class C alleles that we tested partially complemented the eyeless phenotype when placed in *trans* to Class A alleles, consistent with enhancer action in *trans* being generally permissible for both Class B and Class C alleles of *eya* (Figs 3 and S2). Notably, Class B alleles consistently showed higher levels of complementation relative to Class C alleles in both *eya*^*1*^ and *eya*^*2*^ backgrounds; specifically, while there was no significant difference in the numbers of ommatidia of flies carrying *eya*^*3*^ and *eya*^*4*^ alleles as *trans*-heterozygotes with *eya*^*1*^, each had a significantly higher ommatidia count than the Class C alleles in combination with *eya*^*1*^ (adjusted p < 0.05, Kruskal-Wallis test with Dunn’s multiple comparisons test). The same was true of ommatidia counts of Class B and Class C alleles in combination with the Class A allele *eya*^*2*^ with the exception of one comparison (*eya*^*3*^*/eya*^*2*^ vs *eya*^*cliIID*^*/eya*^*2*^, adjusted p = 0.17). Thus, our data support that the strength of complementation between Class A and Class B alleles is consistently greater than that between Class A and Class C alleles.

**Fig 4:**
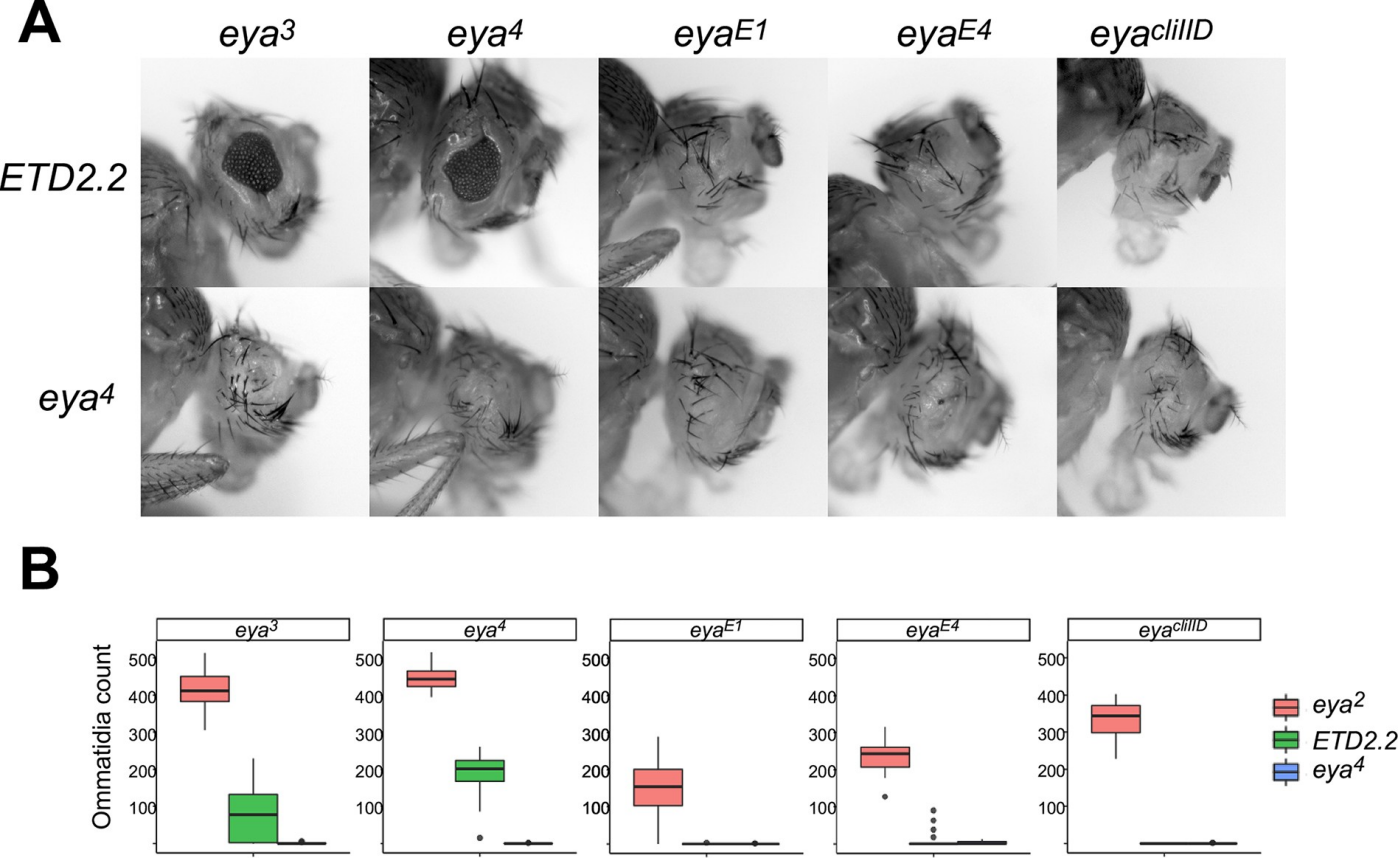
Transvection at *eya* via enhancer action in *trans* requires somatic homolog pairing. **A**, eyes of representative flies carrying the allele indicated at left in *trans* to the Class B or Class C allele indicated above. ETD2.2 is a transvection-disrupting rearrangement of a second chromosome carrying *eya*^*2*^. **B**, quantification of eye development for flies carrying the indicated alleles. Approximately 20 eyes from 10 flies were scored for each genotype. Note that the data for eya^2^ crosses are identical to those presented in Fig 3.

To confirm that the complementation observed between Class A and Class B or C alleles was due to enhancer action in *trans*, we took advantage of a chromosomal rearrangement of the *eya*^*2*^ allele that was previously shown to disrupt transvection between *eya*^*2*^ and *eya*^*4*^ [27]. All Class B and Class C alleles failed to complement the rearranged *eya*^*2*^ allele *ETD2*.*2* to the extent that they complemented a structurally wild type *eya*^*2*^ chromosome, confirming that intragenic complementation is indeed pairing-dependent (Fig 4). Furthermore, Class B alleles showed partial complementation in combination with ETD2.2, with an average count of 133.3 ± 85.1 ommatidia per eye, whereas Class C alleles showed a near complete failure of complementation with ETD2.2, with the majority of flies being completely eyeless (Fig 4). Importantly, all Class B and Class C alleles completely fail to complement the eyeless phenotype of the Class B allele *eya*^*4*^ (Fig 4A), indicating that differences in transvection observed for Class B vs Class C are unlikely to be due to general differences in the penetrance or expressivity of the eyeless phenotype between the two allele classes [9, 27]. In sum, Class B alleles, which carry insertions near the promoter of the *eya-B* transcript, support higher levels of transvection with Class A alleles than do Class C alleles, which have no disruptions near the B-transcript promoter.

As an additional test, we assessed the impact of mutations in *Cap-H2*, a component of the Condensin II complex, on intragenic complementation between alleles of *eya*. Loss of *Cap-H2* has been shown to increase levels of somatic homolog pairing, which can result in elevated levels of transvection relative to a wild type *Cap-H2* background [29, 30]. Indeed, flies of genotype *eya*^*2*^/*eya*^*4*^; *Cap-H2*^*0019*^/*Cap-H2*^*5163*^ have a significantly greater number of ommatidia than *eya*^*2*^/*eya*^*4*^ flies in wild-type *Cap-H2* backgrounds (p = 0.02, Mann-Whitney test) (S3 Fig), adding further evidence that intragenic complementation at the *eya* locus is due to transvection.

### Enhancer action in *trans* reveals plasticity in enhancer-promoter specificity

**Fig 5:**
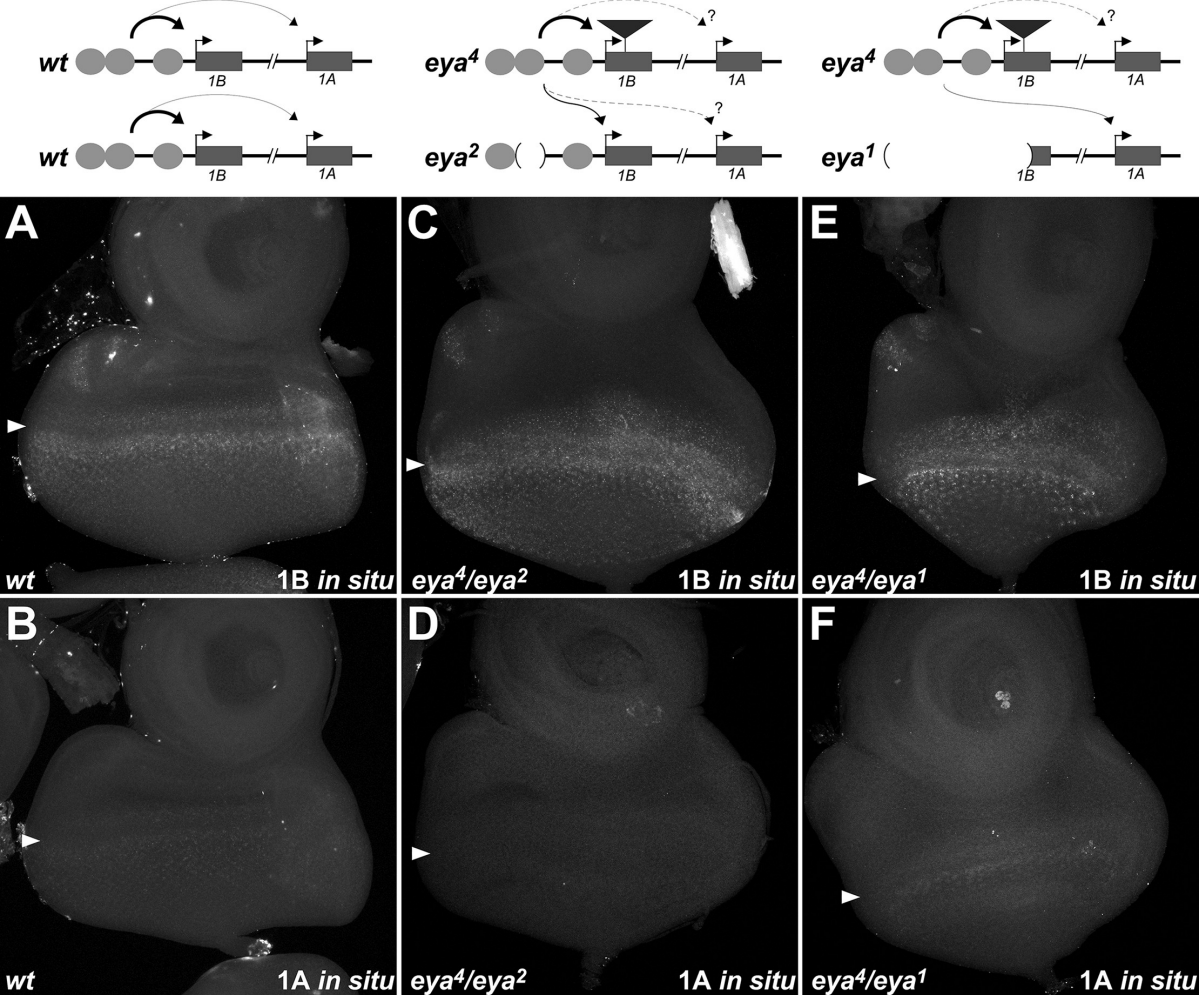
Plasticity in promoter use for enhancer action in *trans*. **A-F**, third instar eye-antennal discs from wild type (**A, B**), *eya*^*4*^/*eya*^*2*^ (**C, D**), or *eya*^*4*^/*eya*^*1*^ (**E, F**) larvae. Discs were subjected to i*n situ* hybridization using probes specific to exon 1B (**A, C, E**) or exon 1A (**B, D, F**). Arrowheads indicate the approximate position of the morphogenetic furrow. Robust staining is seen for exon 1B in all cases; Exon 1A shows weak staining in wild type, is generally undetectable above background staining in *eya*^*4*^/*eya*^*2*^, and appears prevalent in *eya*^*4*^/*eya*^*1*^. Schematic diagrams (above) indicate likely promoter usage in each genotype.

Prior analyses have demonstrated that, whereas expression during embryogenesis is specific to the *eya-A* promoter, both the *eya-A* and *eya-B* promoters are active in the developing eye disc [20, 26]. Furthermore, qRT-PCR analysis supports that the eye-specific enhancers upstream of the *eya-B* promoter activate transcription of both transcript types [26]. To better understand transcript-specific expression in the developing eye, we performed *in situ* hybridization on wild type third instar larval eye discs using probes specific to the first exons of either the *eya-A* or *eya-B* transcript. Analysis of the *eya-B* isoform showed robust expression in progenitor cells anterior to the morphogenetic furrow and in differentiating cells immediately posterior the furrow, with lower levels of expression observed in more mature ommatidial clusters toward the posterior of the disc (Fig 5A). Expression is also seen in the developing ocelli, consistent with a requirement for *eya* in ocelli development [16]. The pattern of staining for the *eya-A* transcript appears similar to that of the *eya-B* transcript, with highest expression seen immediately anterior and posterior to the morphogenetic furrow (Fig 5B). However, the signal for the *eya-A* transcript is barely detectable above background fluorescence, suggesting that the eye-specific enhancers of *eya* act predominantly on the *eya-B* promoter in the developing eye, and only act weakly on the *eya-A* promoter.

The alleles *eya*^*3*^ and *eya*^*4*^ are caused by retrotransposon insertions in the first exon of *eya-B* (Exon 1B), and do not directly impact the *eya-A* transcript [24]. Furthermore, *in situ* hybridization demonstrates that the retroelement in *eya*^*4*^ is transcribed in an *eya*-like pattern, reflecting that the eye enhancers are functional in this allele [24]. Two plausible models could account for the lack of eye development in *eya*^*3*^ and *eya*^*4*^ flies: first, assuming that the *eya-B* transcript is rendered non-functional by the retroelement insertions, production of functional mRNA solely from the *eya-A* promoter could be insufficient to generate eye tissue. Alternatively, it could be that retrotransposon insertion into Exon 1B decreases or prevents communication between eye-specific enhancers and the *eya-A* promoter, resulting in loss of *eya-A* transcription and leaving only the non-functional *eya-B* transcript. To assess these models and thereby gain a better understanding of transcript usage in the developing eye, we employed isoform-specific *in situ* hybridization in discs that display enhancer action in *trans*. In *eya*^*4*^*/eya*^*2*^ discs, we observe robust expression of *eya-B*, likely reflecting strong activation of the retrotransposon-carrying Exon 1B in *cis* to the functional enhancers of the *eya*^*4*^ chromosome in addition to *trans*-activation of the functional Exon 1B on the *eya*^*2*^ chromosome (Fig 5C). However, levels of the *eya-A* transcript appear strongly decreased in *eya*^*4*^*/eya*^*2*^ discs (no detectable signal in 6/7 discs scored) (Fig 5D), suggesting that the retrotransposon insertion into Exon 1B significantly decreases transcription from the *eya-A* promoter, and that the *eya-B* promoter is the preferred target in *cis* and in *trans* to the eye-specific enhancers.

In contrast to the small enhancer deletion of the *eya*^*2*^ allele, the *eya*^*1*^ deletion removes enhancer sequences and the *eya-B* promoter [26]; thus, in *eya*^*4*^*/eya*^*1*^ discs, functional *eya-B* transcript cannot be generated from either chromosome. In contrast to our observations in *eya*^*4*^*/eya*^*2*^ discs, we easily detect RNA signal for the *eya-A* transcript in *eya*^*4*^*/eya*^*1*^ discs (7/7 discs scored), consistent with a model wherein the *eya-A* promoter is *trans*-activated by the functional enhancers of the *eya*^*4*^ chromosome in this background (Fig 5E and 5F). To further support a difference in promoter usage in *eya*^*4*^*/eya*^*2*^ *vs*. *eya*^*4*^*/eya*^*1*^ discs, we employed isoform-specific quantitative RT-PCR on eye-antennal discs from these genotypes and compared the levels of *eya-A* transcripts relative to those of *eya-B*. Notably, *eya-A* transcript levels in *eya*^*4*^*/eya*^*2*^ discs drop to 56% (95% CI 52.6%-59.9%, n = 3 biological replicates) when compared to transcripts from *eya*^*4*^*/eya*^*1*^ discs, further supporting decreased expression of *eya-A* relative to *eya-B* in *eya*^*4*^*/eya*^*2*^ discs. In sum, our data demonstrate that the eye-specific *eya* enhancers show a preference for the *eya-B* promoter, and support that the loss of eye development in *eya*^*3*^ and *eya*^*4*^ flies involves a reduction in activation of the *eya-A* transcript in addition to the insertional disruption of the *eya-B* transcript. Furthermore, our data suggest that, in the absence of a functional *eya-B* promoter in *trans*, the enhancers can switch their specificity to *trans*-activate the *eya-A* promoter in order to produce eye tissue.

### Plasticity in enhancer-promoter specificity in *cis* revealed by deletion of a preferred promoter target

**Fig 6:**
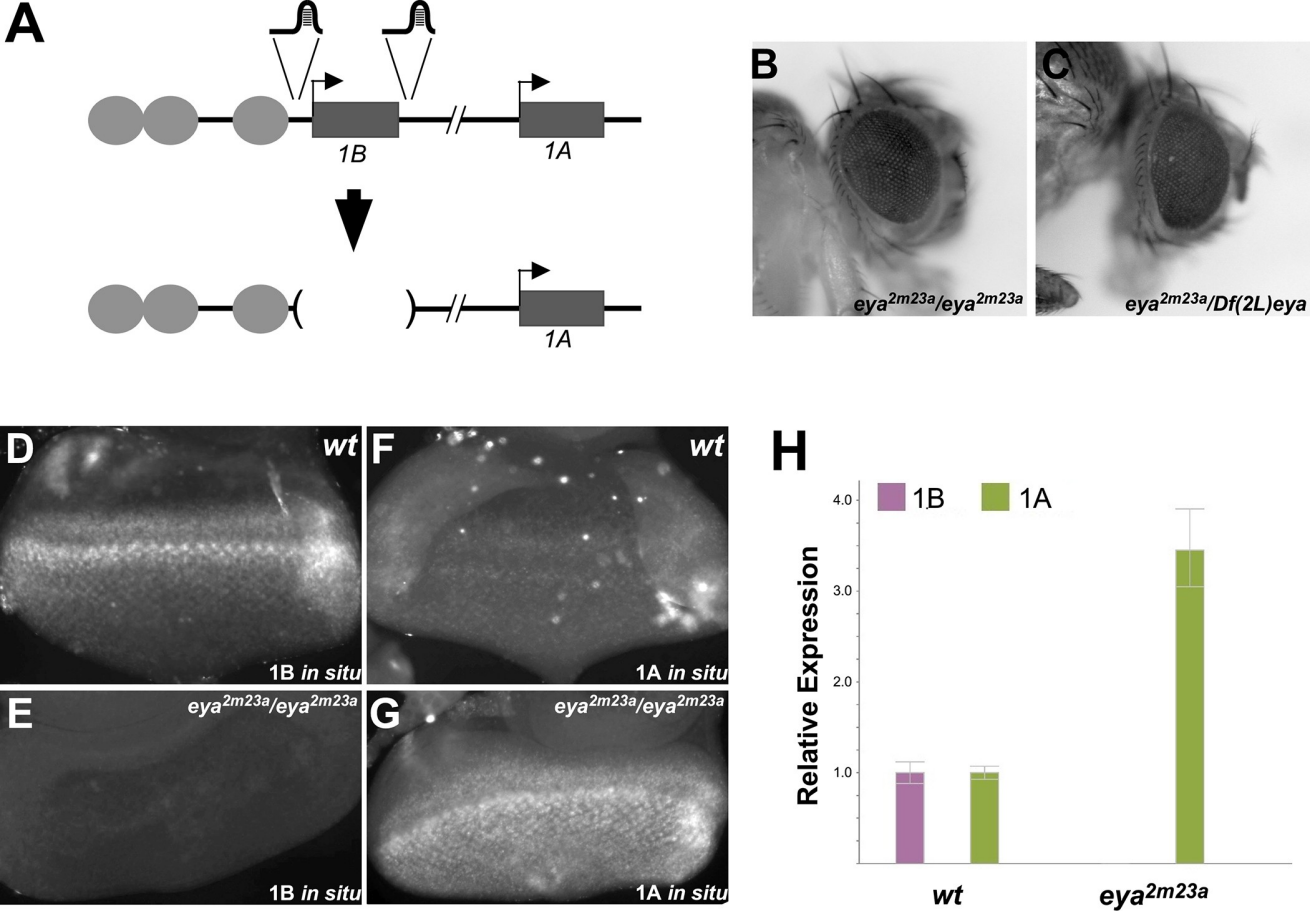
Deletion of the *eya-B* promoter causes a switch in specificity of eye-specific enhancers to the *eya-A* promoter. **A**, strategy for sgRNA design to delete the core promoter and first exon of the *eya-B* transcript using CRISPR/Cas9. **B-C**, eyes of representative flies homozygous (**B**) or hemizygous (**C**) for the allele *eya*^*2m23a*^, which carries the deletion indicated in part A. Eyes are nearly identical to wild type. **D-G**, third instar eye-antennal discs from wild type (**D, E**), or *eya*^*2m23a*^/*eya*^*2m23a*^ (**F, G**) larvae subjected to i*n situ* hybridization using probes specific to exon 1B (**D, F**) or exon 1A (**E, G**). Loss of exon 1B in *eya*^*2m23a*^ flies leads to robust upregulation of the *eya-A* transcript. **H**, quantitative RT-PCR on cDNA isolated from wild type (left) or homozygous *eya*^*2m23a*^ (right) third instar eye-antennal discs using primers specific for exon 1B or exon 1A. Deletion of exon 1B results in a roughly 3.5-fold increase in transcripts from the A promoter.

To further assess the requirement for the *eya-B* transcript in eye development, we used CRISPR-Cas9 to completely remove Exon 1B and its associated core promoter from the genome (Fig 6A). Synthetic guide RNAs designed to flank Exon 1B and under the control of the U6 promoter were injected into embryos carrying a source of Cas9, and the progeny of the resulting flies were screened via PCR for the expected deletion, resulting in four independent mutants lacking exon 1B (see Materials and Methods). All four mutant alleles are viable and fertile as homozygotes, consistent with the proposed eye-specific role for Exon 1B. Surprisingly, all mutants lacking Exon 1B develop near-wild type eyes as either homozygotes or in combination with *Df(2L)eya* (Fig 6B and 6C). To address eye-specific promoter usage in these mutants, we performed isoform-specific *in situ* hybridization on third instar larval eye-antennal discs that were homozygous for the Exon 1B deletion. We observed no signal above background for the *eya-B* probe, confirming that the induced deletion prevents transcription of these sequences (Fig 6D and 6E). Remarkably, staining for the *eya-A* transcript shows robust signal in a pattern similar to that previously observed for *eya-B*, demonstrating that the loss of the *eya-B* promoter results in elevated activation of *eya-A* transcription (Fig 6F and 6G). To further support this observation, we used quantitative RT-PCR to measure levels of *eya-A* and *eya-B* expression in wild type and exon 1B-deleted mutant eye-antennal discs (Fig 6H). Consistent with our *in situ* data, we observed a 3-fold increase in expression of *eya-A*, and a complete loss of *eya-B*, in the mutant discs. Thus, our data support a model wherein wild type eye development relies primarily on activation of the *eya-B* promoter; in the absence of this promoter and its associated first exon, enhancers shift their specificity to the more distal promoter associated with the *eya-A* transcript, with near complete compensatory expression to support eye development.

### The *eya*^*cs*^ allele demonstrates both active and repressive *trans*-interactions

**Fig 7:**
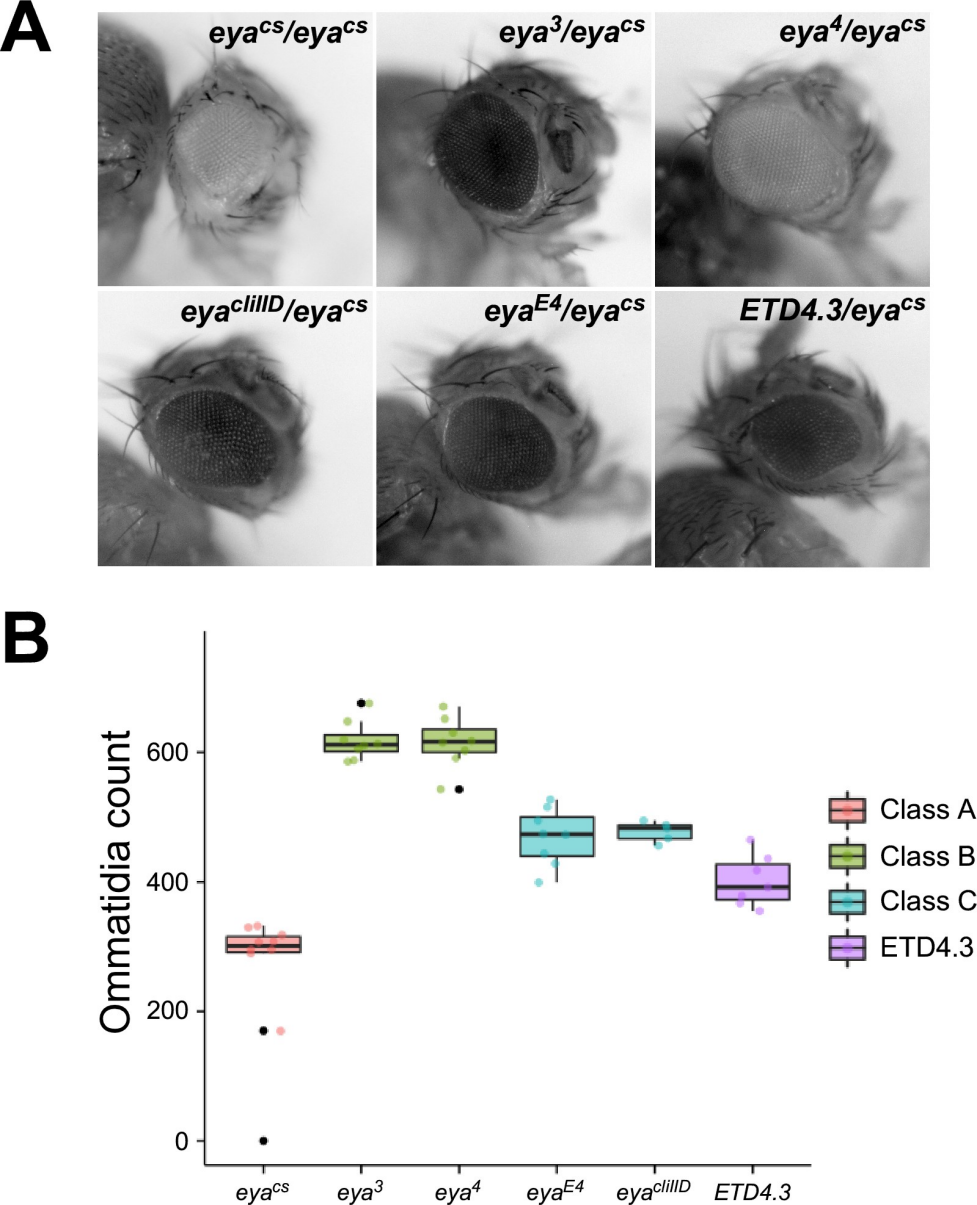
The hypomorphic Class A allele *eya*^*cs*^ can participate in enhancer action in *trans*. **A**, eyes of representative flies carrying the indicated genotypes. ETD4.3 is a transvection-disrupting rearrangement of a second chromosome carrying *eya*^*4*^. **B**, quantification of eye development for flies carrying the indicated alleles in *trans* to *eya*^*cs*^. Consistent with enhancer action in *trans*, increased eye development is observed when *eya*^*cs*^ is placed in *trans* to Class B or Class C alleles relative to *eya*^*cs*^ homozygotes, and the increase is disrupted by the *eya*^*4*^ rearrangement ETD4.3 (compare column 3 to column 6).

The Class A allele *eya*^*cs*^ is homozygous viable and hypomorphic, with *eya*^*cs*^/*eya*^*cs*^ flies showing a reduced adult eye phenotype with variable expressivity (Fig 7A and 7B). Sequence analysis showed that *eya*^*cs*^ carries a 115 bp deletion from -806 to -691 relative to the TSS of the B transcript, which is nested within the enhancer deleted by the *eya*^*2*^ allele (-896 to -577) (Fig 2A). To assess whether *eya*^*cs*^ can support enhancer action in *trans*, we created flies with *eya*^*cs*^ on one homolog and various Class B or Class C alleles on the other homolog as we had previously done with the Class A alleles *eya*^*1*^ and *eya*^*2*^. Flies carrying *trans*-heterozygous combinations of *eya*^*cs*^ and Class B or Class C alleles show greater numbers of ommatidia than *eya*^*cs*^ homozygotes, comfirming increased expression of *eya* (Fig 7A and 7B). As observed for other Class A alleles, the strength of transvection is higher when *eya*^*cs*^ is in *trans* to Class B alleles relative to Class C alleles, and a transvection-disrupting rearrangement of the chromosome carrying *eya*^*4*^ [27] shows reduced complementation relative to a structurally wild type chromosome carrying *eya*^*4*^, supporting that the observed complementation between *eya*^*cs*^ and Class B and Class C alleles are pairing-dependent (Fig 7A and 7B). Thus, the hypomorphic Class A allele *eya*^*cs*^ can participate in enhancer action in *trans*.

**Fig 8:**
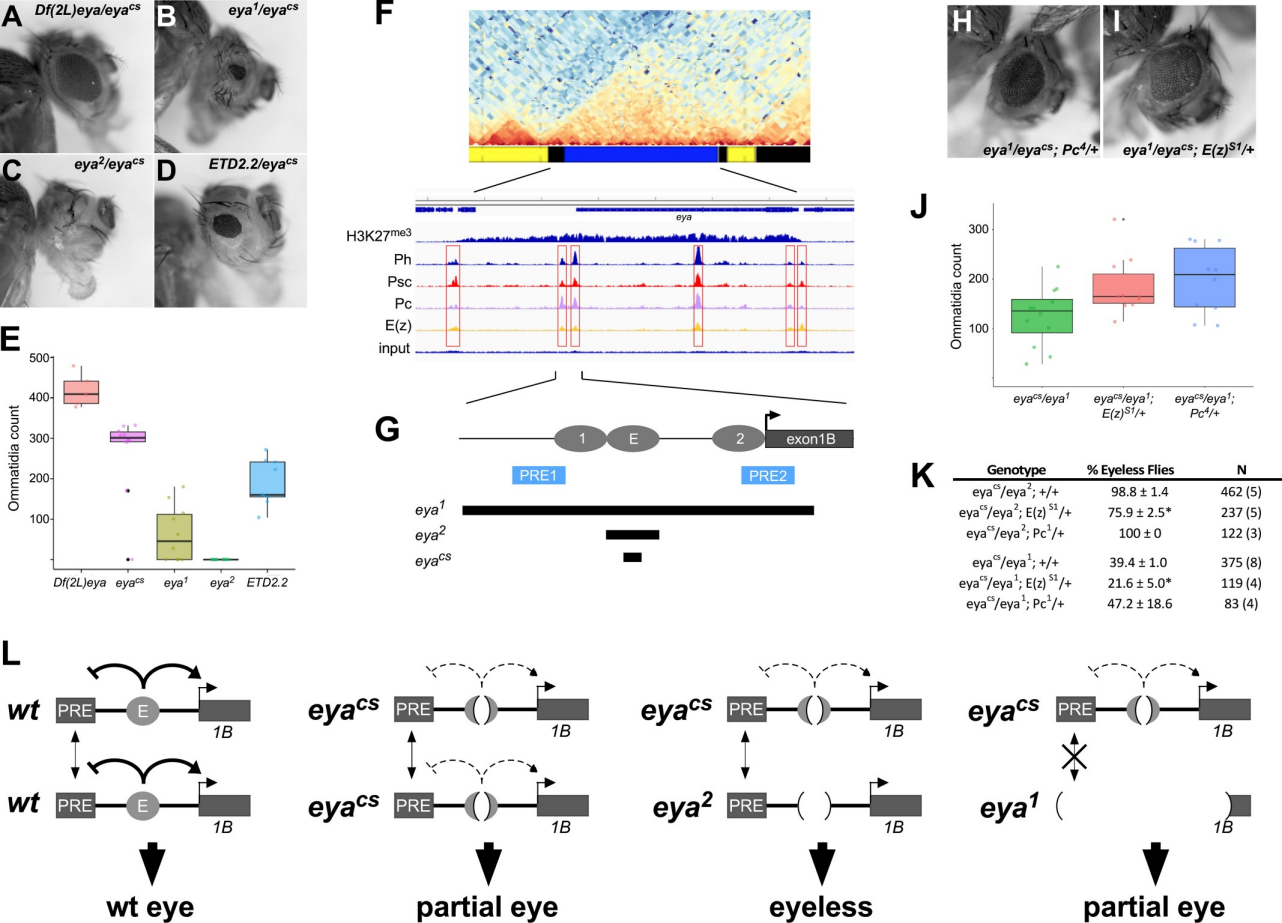
Pairing sensitive silencing between Class A alleles reflects a direct role for PcG proteins in regulating *eya*. **A-D**, eyes of representative flies carrying the indicated genotypes. *ETD2*.*2* is a transvection-disrupting rearrangement of a second chromosome carrying *eya*^*2*^. **E**, quantification of eye development for flies carrying the indicated alleles in *trans* to *eya*^*cs*^. Consistent with pairing-sensitive silencing, decreased eye development is observed when *eya*^*cs*^ is placed in *trans* to other Class A alleles relative to *eya*^*cs*^ homozygotes, and the decrease is disrupted by the *eya*^*2*^ rearrangement ETD2.2 (compare column 4 to column 5). Furthermore, 98.8% of *eya*^*cs*^/*eya*^*2*^ flies are completely eyeless (n = 462; see Panel K), but all (n = 120) *eya*^*cs*^/*ETD2*.*2* flies develop scorable eye tissue. **F**, genomic features of the *eya* locus (note that genomic maps are reversed relative to the reference sequence.) Top, Hi-C contact map showing TAD structure, with chromatin color map below. The *eya* locus occupies a TAD that is primarily blue chromatin, indicative of Polycomb silencing. Below, ChIP-seq peaks for K27-trimethylated histone H3 and individual PcG proteins as assayed from larval disc tissues. Six putative PREs (red boxes) are indicated by the pattern of peaks. **G**, schematic showing positions of eye-specific enhancers of putative PREs upstream of the *eya* B promoter. Bars below the schematic indicate the deletions carried by Class A alleles. **H-I**, eyes of representative flies carrying the indicated genotypes. **J**, quantification of eye development for flies carrying the indicated genotypes. Completely eyeless flies were not scored in this analysis. Significant increases in the ommatidia count are observed for *eya*^*cs*^*/eya*^*1*^*; E(z)*^*S1*^*/+* (p = 0.03, Mann-Whitney test) and *eya*^*cs*^*/eya*^*1*^*; Pc*^*4*^*/+* (p = 0.04) relative to *eya*^*cs*^*/eya*^*1*^*; +/+*. **K**, scoring of flies that have at least one eye of any size in the indicated genotypes. Asterisks indicates significant difference relative to *eya*^*cs*^*/eya*^*2*^*; +/+* (p = 0.008, Mann-Whitney U test) or *eya*^*cs*^*/eya*^*1*^*; +/+* (p = 0.03). “N” indicates the number of flies scored, with the number of separate vials in parentheses. **L**, Models for interactions between eye-specific enhancers and putative PREs. For simplicity, only the E enhancer (required for initiating eye development) and its neighboring PRE are shown; we expect that other local PREs behave similarly. In wild type, we propose that the enhancer has dual roles to block silencing by PREs through an unknown mechanism and to activate transcription primarily from the *eya-B* promoter. The *eya*^*cs*^ allele retains partial enhancer activity, whereas the *eya*^*2*^ allele has no detectable enhancer activity; in *eya*^*cs*^*/eya*^*2*^ flies, pairing between homologous PREs (dual arrows) increases their capacity to silence, resulting in an eyeless phenotype in the paired state, but a partial eye when unpaired by chromosomal rearrangements. Since *eya*^*1*^ deletes some of the putative PREs, pairing-dependent silencing is reduced, and the *eya*^*cs*^*/eya*^*1*^ phenotype is less severe than that of *eya*^*cs*^*/eya*^*2*^.

In establishing *eya*^*cs*^ as a Class A allele, we were surprised to find that the eye phenotypes of flies carrying *eya*^*cs*^ *trans*-heterozygous with the other Class A alleles are more severe than those of *eya*^*cs*^ homozygotes, with *eya*^*cs*^*/eya*^*2*^ having a more severe phenotype than *eya*^*cs*^*/eya*^*1*^ (Fig 8A–8E). Furthermore, the eye phenotype of flies carrying *eya*^*cs*^ *trans*-heterozygous with *Df(2L)eya*, a large deficiency spanning the entire *eya* locus, does not show an increased severity relative to *eya*^*cs*^ homozygotes, but instead shows a more moderate phenotype (Fig 8A and 8E). Thus, *eya*^*cs*^ shows repressive *trans*-interactions with the small deletions carried by other Class A alleles, but not with a large deletion. To determine whether repressive *trans*-interactions involving *eya*^*cs*^ are pairing-dependent, we created *trans*-heterozygotes between *eya*^*cs*^ and the rearranged *eya*^*2*^ allele *ETD2*.*2* (Fig 8D and 8E). Notably, the disruption of pairing between *eya* alleles caused by the rearrangement carried by *ETD2*.*2* restored partial eye development in these flies, indicating that the repression of *eya* by other Class A alleles is pairing-sensitive.

Previous analyses of pairing-sensitive silencing have revealed a central role for *PcG* genes [7]. Specifically, known cases of pairing-sensitive silencing are caused by pairing-dependent interactions between PREs on homologous chromosomes, which is thought to augment the recruitment and/or silencing capacity of PcG complexes relative to those at unpaired PREs. To address whether PcG genes may function in pairing-sensitive silencing at the *eya* locus, we first assessed ChIP-seq binding profiles of key PcG proteins and the histone H3K27^me3^ mark associated with Polycomb repressive domains (Fig 8F). Indeed, the compartmental domain occupied by *eya* is rich in H3K27^me3^ in third instar larval disc tissue and in cultured *Drosophila* cells [31–33], and carries several putative PREs indicated by peaks of PcG proteins Ph, Psc, Pc, and E(z) [33]. Two putative PREs are located upstream of the eye-specific *eya-B* promoter and partially overlap the known eye-specific enhancers [26] (Fig 8F and 8G). Notably, the *eya*^*2*^ deletion is predicted to leave all putative PREs intact, whereas the *eya*^*1*^ deletion removes the two putative PREs that are upstream of the *eya-B* promoter (Fig 8G).

To provide genetic evidence for a role for PcG proteins in pairing-dependent silencing at *eya*, we used two approaches to assess eye development in backgrounds with reduced expression of two key PcG components, E(z) and Pc. First, we scored the number of ommatidia in developed adult eyes (excluding eyeless flies) for *eya*^*cs*^*/eya*^*1*^ *trans*-heterozygous flies with and without reduced PcG gene dosage. In this assay, mutations in both *E(z)* and *Pc* act as dominant suppressors of pairing-sensitive silencing (Fig 8H–8J), resulting in greater numbers of ommatidia relative to *eya*^*cs*^*/eya*^*1*^ flies with wild type PcG dosage. Secondly, we compared percentages of total adult flies that were completely eyeless; in an *eya*^*cs*^*/eya*^*2*^ background, nearly all (98.8 ± 1.4%) adults completely lack eyes, but loss of a single functional copy of *E(z)* suppressed the number of eyeless flies to 75.9 ± 2.5% (p = 0.008, Mann-Whitney U test) (Fig 8H). A similar effect is observed in an *eya*^*cs*^*/eya*^*1*^ background (p = 0.02), but not when a functional copy of *Pc* was removed from either *eya*^*cs*^*/eya*^*1*^ or *eya*^*cs*^*/eya*^*2*^ flies (Fig 8H), perhaps indicating a greater sensitivity for *E(z)* relative to *Pc* function at an early stage of eye specification. In sum, our genetic data and genomic analysis support a role for PcG genes in pairing-sensitive silencing at *eya*.

Based on our data, we favor a model wherein the *eya* eye-specific enhancers play dual roles, activating transcription of the *eya-B* and *eya-A* promoters and opposing the silencing activity of PREs in the domain occupied by *eya* (Fig 8L). In flies carrying the *eya*^*cs*^ deletion, one or both of these activities is partially compromised, resulting in reduced eye growth; when *eya*^*cs*^ is placed in *trans* to the *eya*^*2*^ allele, enhancer activity is further suppressed while pairing between homologous PREs strengthens their silencing capacity, resulting in complete loss of eye tissue. However, when *eya*^*cs*^ is placed in *trans* with *eya*^*1*^ or larger deletions, or with a rearranged *eya*^*2*^ allele, pairing of some homologous PREs is lost, resulting in re-establishment of partial eye development. In sum, our data indicate an important role for PcG genes in regulating a critical eye determining gene.

## Discussion

The proper expression of developmental genes represents a complex interplay of interactions between regulatory sequences that include enhancers, promoters, and silencers such as PREs. Growing evidence supports that regulatory interactions depend upon the positions of DNA elements in three-dimensional space and can be influenced by potential competing interactions with neighboring DNA elements. Due to the complexity of interactions in wild type animals, mutations in regulatory sequences are critical in helping us to better understand how the interaction landscape is assembled via the roles played by individual elements. Furthermore, the study of *trans*-interactions in *Drosophila* can uncover aspects of gene regulation that are masked or otherwise challenging to understand in other contexts. Here, by examining *cis*- and *trans*-interactions between various classes of *eya* mutants, we demonstrate a hierarchical set of interactions that shed insight on how enhancers may choose between multiple promoter targets, and suggest an important antagonistic relationship between transcriptional activation by *eya* enhancers and silencing by local PREs.

Our study was motivated in part by a desire to better understand how gene expression is influenced by *trans*-interactions, or transvection, in *Drosophila*. Our data support that the “A, B, C” allele classification put forth by Morris et al. [9] accurately predicts patterns of complementation via enhancer action in *trans*. Specifically, complementation of Class A alleles by Class B alleles is consistently higher than that by Class C alleles at both *yellow* and *eya*. In some Class B alleles of *yellow*, the core promoter is directly deleted or otherwise mutated, which has led to a model wherein competition by the functional *cis*-promoters in Class C alleles is a likely explanation for weaker transvection relative to Class B [9, 10]. Other Class B alleles of *yellow*, and the Class B *eya* alleles *eya*^*3*^ and *eya*^*4*^, are instead characterized by transposon insertions into the 5’UTR downstream of the promoter, which may cause changes in topology that alter the balance of enhancer-promoter interaction in *cis* vs *trans*, although direct evidence for this model is lacking. It should also be noted that variation in strength of enhancer action in *trans* can be seen within Classes; for example, our data consistently show higher levels of complementation by the Class A allele *eya*^*2*^ relative to that by *eya*^*1*^. This could reflect differences in the specific enhancer elements affected by each deletion, or, alternatively, could result from the availability of the preferred *eya-B* promoter on the *eya*^*2*^ chromsome vs. its absence on the *eya*^*1*^ chromosome (Fig 5). Similar to these observations, several alleles of *Malic Enzyme* (*Men*) would be categorized as Class B since they each carry a promoter deletion, yet they show highly varying levels of *trans*-activity [34]. Thus, The ABC classes of alleles reflect rough categorizations with respect to an allele’s participation in enhancer action in *trans*.

To date, transvection effects have been observed for relatively few genes in the *Drosophila* genome, although transgenic studies using diverse enhancers and genomic locations suggest that enhancer action in *trans* is widely supported [10, 14, 35–37]. However, we note that genetic interactions consistent with enhancer action in *trans* have also been reported for two other RDN members, *so* and *eyeless* (*ey*) [38–40]. It may be purely coincidental that a substantial proportion of RDN genes show transvection effects, or, alternatively, somatic homolog pairing and transvection may be of particular importance to gene regulation in the developing eye. Consistent with the latter hypothesis, *trans*-interactions at the *spineless* (*ss*) locus were found to be critical for later fate specifications of photoreceptor types [41]. It will be interesting to explore the potential for *trans*-interactions at other genes required for eye development to further test this hypothesis.

### Plasticity of enhancer-promoter specificity at the *eya* locus

The presence of multiple *eya* promoters likely reflects an ancient promoter duplication event, which, like gene duplications, can lead to varying degrees of functional redundancy or sub-functionalization between the alternate TSS [42]. Promoter duplication appears to be widespread in the *Drosophila* genome; genome-wide mapping of TSS shows that approximately 27% of mapped genes can initiate transcription via two or more promoters, with an average number of 1.4 promoters per gene across all mapped TSS [43]. Comparison to genomes of other *Drosophila* species suggests that a promoter duplication at *eya* was a relatively recent event, with evidence of two TSS in the *D*. *melanogaster*, *D*. *simulans*, and *D*. *ananassae* genomes, but not in those of *D*. *pseudoobscura* or *D*. *virilis* [44, 45].

Prior analyses at *eya* suggest that the *eya-A* and *eya-B* promoters have undergone some degree of sub-functionalization in *D*. *melanogaster*, with the *eya-B* promoter being active primarily within the developing eye disc and the *eya-A* promoter being more broadly expressed across multiple tissues [20]. Our data support the prior finding that the *eya-B* promoter is the preferred target of the eye-specific *eya* enhancers, which are primarily located just upstream of the *eya-B* promoter, but roughly 10 kb from the *eya-A* promoter [26]. These observations suggest a simple model wherein specificity can be dictated by relative position; for some enhancers, activity may be highest on nearby promoters, with less activity on more distal promoters. However, our data support that this model is largely driven by promoter competition at the *eya* locus such that, in the absence of the *eya-B* promoter, the *eya-A* promoter becomes a “preferred” target and is highly active. Interestingly, ChIP-seq analysis using antibodies to the insulator protein *su(Hw)* in embryos suggests the presence of an insulator element in the *eya-B* first intron that would be predicted to disrupt communication between the eye-specific enhancers and the *eya-A* promoter [46]. Based on our observations, it is unlikely that this candidate insulator is active in the developing eye disc.

Our data regarding promoter competition and enhancer-promoter proximity is consistent with prior observations where a nearby promoter is preferred to one that is more distal [47–50], and may therefore be generalizable to many enhancers. As a potential caveat in interpreting our data, two other DNA fragments that map close to the *eya-A* promoter support some degree of transgene expression in the late developing eye disc [26]. These candidate enhancers are not themselves sufficient to rescue eye phenotypes, and are therefore of unknown functional relevance *in vivo*, but we cannot exclude the possibility that their activity changes in some way upon deletion of the *eya-B* promoter such that they play a role in the upregulation of *eya-A* transcription observed in this background. Finally, we note that the current genome annotation supports evidence for a third eya TSS, defining an *eya-C* transcript that initiates further downstream from the *eya-A* TSS and is predicted to produce a truncated protein product [45, 51–52]. The biological relevance of this potential promoter and its relationship to the *eya-A* and *eya-B* transcripts is as yet unclear.

### Pairing-sensitive silencing at *eya* indicates *eya* expression is a balance of activating and repressive signals

Our observation of pairing sensitive silencing of *eya* suggests a direct role for PcG genes in regulating *eya* expression. In support of this hypothesis, genomic data shows that *eya* is embedded in a domain of H3K27^me3^ in cultured cells, embryos, and third instar disc tissues, and distinct peaks of PcG proteins that are characteristic of PREs are found throughout the *eya* locus [31–33, 53, 54]. Furthermore, reduction in dosage of key PcG proteins, E(z) and Pc, suppresses pairing sensitive silencing of *eya*, providing genetic evidence for a role for PcG proteins in directly regulating *eya* expression.

Recently, Erceg et al [55] characterized hundreds of sequences with overlapping PcG binding and enhancer activity, and showed that these fragments can act as enhancers in some cell types and as silencing PREs in others. Notably, one of the candidate PREs upstream of *eya* overlaps a previously characterized enhancer [26], and the mutations that uncover pairing-sensitive silencing affect sequences in this region. Our data support a model wherein the PRE activity of this region is active in cells outside of the developing eye, silencing *eya*, whereas the PRE silencing activity is overcome in primordial eye cells in order for eye development to proceed. According to this model, H3K27 methylation would be reduced or suppressed by the activity of the *eya* enhancers in primordial eye cells (Fig 8L), although we are unable to observe this directly using existing ChIP-seq data derived from mixed larval tissues. Given that several candidate PREs are found across the locus, it is as yet unclear how these different sequences may cooperate and/or interact to determine the transcriptional state of *eya* in a given tissue.

Interestingly, several other genes of the Retinal Determination Network (RDN) are also characterized by domains of H3K27^me3^ and localized regions of PcG binding in multiple cell types [31–33, 53, 54]. Furthermore, in addition to *eya*, RDN genes *toy* and *dac* have been identified as having overlapping PRE and enhancer sequences, and a neuronal enhancer from the *ey* gene was shown to have enhancer activity in some tissues and PRE activity in others [55], suggesting that direct regulation by PcG proteins could be a common feature of RDN genes. According to this model, PcG proteins would maintain RDN genes in an inactive state in non-eye tissues, whereas activation of RDN gene transcription in the developing eye would rely on the coordinated removal of repressive chromatin marks and simultaneous activation of transcription. Consistent with this hypothesis, ChIP-seq analysis shows that binding of the PcG proteins Pho and Ph at the TSS of the RDN genes *so* and *toy* is higher in haltere tissues (where the RDN genes are inactive) relative to binding in eye tissue, consistent with reduced binding of PcG proteins at PREs of RDN genes in cells with active expression [56]. However, investigations of roles for PcG proteins in the developing eye are complicated by the widespread pleiotropic effects on gene expression caused by PcG mutations combined with the deeply intertwined regulatory network that determines eye cell fates. For example, clonal loss of *E(z)* and *Pc* in cells anterior to the morphogenetic furrow can lead to reduced expression of *eya* and *dachshund* (*dac*), but this is likely due to misexpression of *teashirt*, which can act as a negative regulator of *eya* and *dac* [57, 58]. Similarly, seminal work by Zhu et al. [59] demonstrated a role for PcG proteins in maintaining eye cell fates in the developing eye via the repression of genes that would signal an alternative wing tissue fate. Furthermore, biochemical studies show that Eya protein is a binding partner for Combgap (Cg), a sequence-specific DNA-binding protein that can recruit PcG complexes to PREs, although genetic analyses show that Cg may act in opposition to other PcG complexes in the developing eye [15, 60, 61]. Ultimately, a multifaceted approach involving targeted mutations of individual response elements, combined with transgenic strategies, in backgrounds with altered availability of PcG gene products will likely be required to unravel precise roles for PcG in regulating genes in the developing *Drosophila* eye.

## Materials and methods

### Stocks and fly husbandry

Stocks carrying alleles *eya*^*2*^, *eya*^*3*^, *eya*^*4*^, *eya*^*E1*^, *eya*^*E4*^, *eya*^*D1*^, *eya*^*cs*^, *Df(2L)eya*, *ETD2*.*2* (a chromosome carrying *eya*^*2*^ and the transvection-disrupting inversion *In(2LR)29C;41*), and *ETD4*.*3* (an *eya*^*4*^ background carrying a transvection-disrupting cyclical translocation with new order *T(2; 3; 4) 30A; 101;* 98D) were obtained from Nancy Bonini (Department of Biology, University of Pennsylvania, PA). Stocks carrying *eya*^*D3*^, *eya*^*D6*^, and *eya*^*D7*^ were obtained from Justin Kumar (Department of Biology, Indiana University, IN). Stocks carrying *eya*^*137*.*39*^, *eya*^*117*.*36*^, and *eya*^*7*.*42*^ were provided by Jennifer Jemc Mierisch (Department of Biology, Loyola University, Chicago, IL). A stock carrying *eya*^*54C2*^ was obtained from Denise Montell (Department of Molecular, Cellular, and Developmental Biology, UC Santa Barbara, Santa Barbara, CA). Stocks carrying *eya*^*1*^, *eya*^*EY13242*^, and *eya*^*cliftIID*^ were obtained from the Bloomington Drosophila Stock Center (Indiana University, IN). Stocks carrying *Cap-H2*^*0019*^ and *Cap-H2*^*5163*^ were provided by Giovanni Bosco (Geisel School of Medicine, Dartmouth College, NH). Stocks carrying *Pc*^*1*^ and *E(z)*^*S1*^ (also known as *E(z)*^*60*^) were obtained from Judy Kassis (NIH). All flies were maintained at 25°C in standard 25 mm-diameter vials containing cornmeal, yeast, sugar, and agar medium with p-hydroxybenzoic acid methyl ester to prevent mold [10].

To assess adult eye development, crosses were established between 1–4 males and 2–5 virgin females of the selected genotypes. Progeny flies were collected 1 to 5 days post-eclosion and frozen for preservation. Fly eyes were imaged using a Canon EOS Rebel Tli digital camera mounted on a Leica MZ7.5 stereomicroscope. For each eye, the number of ommatidia was scored manually from the digital images. Mean count data and standard deviations for crosses examining enhancer action in *trans* are presented in S3 Table as well as the main text figures. Statistical comparisons were made using Graphpad Prism or R.

Backgrounds carrying *PcG* mutations occasionally showed suppression of pairing sensitive silencing in late-eclosing flies from vials that were overcrowded, which is similar to observations of sex comb phenotypes induced by other *PcG* mutations [62]. We did not observe evidence of changes in severity of phenotype for other allelic combinations of *eya* according to eclosion time or crowding. Nevertheless, we avoided overcrowding and did not score flies beyond day 5 of eclosion.

### Identification of molecular lesions in eya alleles

Strategies and analysis for the identification of molecular lesions in alleles of *eya* are detailed in S1 File.

### RNA *in situ* hybridization

To create transcript-specific RNA probes, exon 1 of the *eya-A* transcript and exon 1 of the *eya-B* transcript were each amplified from genomic DNA using primer pairs eyaISA1F/eyaISA1R and eyaISB1F/eyaISB1R, respectively (Primer sequences are provided in S1 Table). For both primer pairs, the reverse primer included a 5’ extension carrying the promoter for T7 RNA polymerase. PCR products were purified using a PCR Purification kit (Qiagen), and 1 μg of each PCR product was used as a template to create digoxygenin-labeled RNA probes using a Dig RNA Labelling Kit (Roche). The products of the reaction were ethanol precipitated and resuspended in 250 μl of 50% formamide/50% TE with 0.1% Tween-20.

For *in situ* hybridization, eye-antennal discs were dissected in PBS, transferred to a 1.5ml microcentrifuge tube, fixed in 4% formaldehyde in PBS on ice for 20 minutes, then fixed further in 4% formaldehyde/PBS with 0.6% Triton X-100 at room temperature for 20 minutes. After washing in PBS + 0.6% Triton X-100 (3 x 5 min), discs were rinsed with 50% PBS/50% Hybridization Buffer (HB, 50% formamide, 2X SSC, 1X Denhardt’s, 250 μg/ml tRNA, 250 μg/ml salmon sperm DNA, 50 μg/ml heparin sulfate, 5% dextran sulfate, 0.1% Tween-20), then pre-hybridized for 1 hour in 500 μl HB at 52°C. Discs were then incubated overnight in HB with a 1:100 dilution of digoxygenin-labeled probe at 52°C with agitation, followed by four changes of wash solution (50% formamide/2xSSC/0.1% Tween-20) over the next 24 hours at 52°C. Discs were rinsed with PBT (PBS + 0.1% Triton X-100), then incubated in PBT for 30 minutes at room temperature. Next, anti-digoxygenin antibody conjugated to horseradish peroxidase (HRP) (Abcam) was added at a dilution of 1:500, and discs were incubated overnight at 4°C. After four 20-minute washes in PBT at room temperature, discs were developed with a TSA-Plus Cy3 detection kit (Perkin Elmer NEL744E001KT), washed in PBT (3 x 5 minutes) and mounted in Fluoromount G (Electron Microscopy Services). Discs were visualized on either a Zeiss Axio Imager.A2 fluorescence microscope with an AxioCam MRm camera and Zen software, or a Leica SP8 confocal microscope with LASX software.

### Quantitative RT-PCR

Assessment of *eya* mRNA levels via quantitative RT-PCR was carried out as previously described [10]. Briefly, for each sample, 20 imaginal discs were dissected from wandering third instar larvae and frozen at -80°C. Tissue homogenization, genomic DNA elimination, and RNA purification were carried out using an RNeasy plus kit (Qiagen) according to the manufacturer’s protocol. PCR was performed on a StepOne Real-Time PCR system (Applied Biosystems) using cDNA diluted 1:5 into SYBR green PCR Mastermix (Applied Biosystems). Primers were designed to specifically amplify the first exon of either the B transcript (primers eyaRTF1 and eyaRTR1) or the A transcript (primers eyaRT_AF1 and eyaRT_AR1). For discs from CRISPR-edited flies, primers RP49-58F and RP49-175R were used to amplify the housekeeping *rp49* cDNA as an internal reference [10]. For discs wherein eye development depended on transvection, RP49 does not present a suitable internal control due to the varying levels of eye tissue relative to the remaining tissues in the disc; in these experiments, the *eya-B* transcript was used as an internal reference for *eya-A* to provide a relative measure of *eya-A*:*eya-B* transcription. Relative levels of transcript were calculated via the ΔΔCt method using StepOne software.

### CRISPR genome editing

To generate a deletion of the eye-specific *eya* exon 1B, primers were designed to create guide RNAs complementary to sequences 64–84 bp upstream of nucleotide +1 of exon 1B and 537–557 bp downstream of the last nucleotide of exon 1B, spanning roughly 1.1 kb of genomic DNA in total (S1 Table). Guide RNAs were cloned into the plasmid pU6-BbsI-chiRNA as previously described [63], and a mixture of two plasmids carrying upstream- and downstream-targeting guide RNAs (250 ng/μl each) was injected into embryos expressing Cas9 under the control of the Actin5C promoter [64] by BestGene, Inc. From 200 injected embryos, 96 G_0_ adults eclosed and were crossed to flies carrying the second chromosome balancer *CyO*. 89 fertile G_0_ adults were subsequently tested for evidence of an exon 1B deletion via PCR using primers eyaCRISPR34check_F and eyaCRISPR34check_R, which flank the region to be deleted via non-homologous end joining of double strand breaks; 68 (76.4%) of these PCRs produced a single 2.5 kb PCR fragment consistent with unmodified wt DNA, whereas 21 (23.6%) of the PCRs produced additional smaller fragments indicative of putative deletions. Of the 21 G_0_ flies carrying candidate deletions, four were found to transmit the deletion through the germline, and isogenic stocks were established from three of these. Sequencing of PCR fragments generated from each stock confirmed that each carries a deletion of roughly 1.1 kb spanning the distance between the two guide RNAs and including exon 1B and its promoter.

## Supporting information

S1 File molecular characterization of *eya* alleles. (DOCX)

S1 Fig The *eya*^*D1*^ allele is a deletion spanning the *eya* locus. Top, *eya*^*D1*^ fails to complement the eye phenotypes of Class A and Class B alleles. Below, strategy for characterization of *eya*^*D1*^ using allele-specific PCR. Primer pairs HopFinder_JTR_F2/HopFinder_JTR_R1 (orange) and eya_P_R1_seq/eya_P_F2_seq (blue) show distinct amplification patterns from *eya*^*2*^ and *eya*^*4*^ chromosomes, respectively. PCR from *eya*^*D1*^*/eya*^*2*^ and *eya*^*D1*^*/eya*^*2*^ *trans*-heterozygotes shows no evidence of amplification from the eyaD1 chromosome.
(TIF)

S2 Fig Class C alleles *eya*^*54C2*^ and *eya*^*D3*^ show patterns of complementation characteristic of other Class C alleles. Counts of ommatidia for *eya*^*54C2*^*/eya*^*2*^ and *eya*^*D3*^*/eya*^*2*^ show complementation consistent with other Class C alleles. Solid line and dark shading represent mean ommatidia counts for *eya*^*2*^ complementation by Class B alleles, dashed line and light shading represent mean ommatidia counts for *eya*^*2*^ complementation by other Class C alleles (see Fig 3). Both alleles completely fail to complement *eya*^*4*^. Data represent n = 20 eyes for each genotype.
(TIF)

S3 Fig Loss of *Cap-H2* enhances transvection of *eya*. Counts of ommatidia are significantly higher in *eya*^*2*^*/eya*^*4*^*; Cap-H2*^*0019*^*/Cap-H2*^*5163*^, carrying a strong *trans*-heterozygous loss of function combination of *Cap-H2* mutations, than in *eya*^*2*^*/eya*^*4*^*; Cap-H2*^*0019*^*/+*, which carries one wild-type copy of *Cap-H2* (p = 0.003, Mann-Whitney test), or *eya*^*2*^*/eya*^*4*^, where both copies of *Cap-H2* are wild type (p = 0.02).
(TIF)

S1 Table Primers used in this study. (DOCX)

S2 Table Single Nucleotide Polymorphisms differentiate *eya*^*E4*^ and *eya*^*54C*^ chromosomes. (DOCX)

S3 Table Summary of crosses supporting enhancer action in *trans*. (DOCX)
