# Supplementary material for: Two modes of transvection at the eyes absent gene of Drosophila demonstrate plasticity in transcriptional regulatory interactions in cis and in trans
Source: PLoS Genet. 2019 May 10;15(5):e1008152. doi: 10.1371/journal.pgen.1008152 (PMC6530868; doi:10.1371/journal.pgen.1008152)
Supplement: S1 File — (DOCX) [file pgen.1008152.s001.docx]

**Molecular characterization of *eya* alleles**

We used a variety of strategies to identify molecular lesions in different alleles of *eya*. Genomic DNA from *eya^54C2^/CyO* and *eya^D3^/CyO* flies was subjected to PCR using primer pairs spanning one or more coding exons, followed by direct sequencing of PCR products. The primer pair eya1F/eyanew2R does not amplify from the *CyO* chromosome due to polymorphisms that overlap the primer sequences, and therefore direct sequencing from PCR fragments generated using this primer pair could be used to determine sequence solely from the *eya* mutant chromosome. For some other primer pairs, we took advantage of a NotI RFLP in exon 3 [1], which allowed us to differentiate PCR fragments that originated from two different chromosomes via NotI digestion prior to sequencing [1]. In other cases, we sequenced PCR fragments without differentiating template specificity, and searched for evidence of position-specific double peaks (indicating a SNV) and/or loss of phasing (indicating an indel) in the chromatogram.

In total, the *eya^54C^* allele was sequenced on both strands through exon 3, encoding the majority of the protein, which uncovered the associated nonsense mutation. The data show that *eya^54C2^* carries the same Q269* nonsense mutation found in the *eya^E4^* allele [1], with specific SNPs elsewhere in the *eya* transcript confirming that these two alleles were isolated independently (S2 Table).

Sequencing of the *eya^D3^* allele showed an indel in exon 5 wherein a TT doublet was replaced with CCCCCCCCCG, causing a frameshift at amino acid 737 and resulting in a C-terminus of 48 random amino acids in place of the usual 23 C-terminal amino acids. The C-terminus of the Eya protein constitutes a domain that shows high conservation between the fly and mouse Eya homologs [1]. The indel creates a new FauI restriction site that is not present in the wild type, which was used to verify the sequencing data via digestion of PCR products (Data not shown). Our analysis showed that the alleles *eya^D3^*, *eya^D6^,* and *eya^D7^*, which are also known as *eya^P3^*, *eya^P6^,* and *eya^P7^* [2, 3], each carry the same indel in exon 5. For all three alleles, we sequenced the coding exons 2-5, the eye-specific exon 1B, and the enhancer region defined by the *eya^2^* deletion, and observed no other obvious causative mutations. These three alleles were generated in the same mutagenesis [2], but the screen was carried out in such a way that each allele should represent an independent mutational event (N. Bonini, personal communication). It thus seems probable that these stocks were mixed at some point in their histories, and we were not able to obtain independent sources for any of the D series alleles. For clarity, we describe only the *eya^D3^* allele in the main text, but our stocks of the *eya^D6^* and *eya^D7^* behave identically.

Genomic DNA from homozygous *eya^cs^* flies was subject to PCR using HopFinder_JTR_F2 and HopFinder_JTR_R1 primers, which flank the known enhancer deletion associated with the *eya^2^* allele. The resulting fragment, which was approximately 100 bp smaller than the PCR fragment generated from control wild type DNA, was sequenced to reveal a 115 bp deletion approximately 700 bp upstream of the Exon 1B promoter.

*Trans*-heteroygotes between *eya^D1^* and either Class A or Class B alleles produces eyeless flies, a behavior characteristic of the large deficiency *Df(2L)eya* (S1 Fig). Similarly, *eya^D1^* is lethal in combination with Class C alleles (data not shown). To support that *eya^D1^* represents a deletion of *eya* regulatory and coding sequences, *trans*-heterozygous *eya^D1^*/*eya^2^* and *eya^D1^*/*eya^4^* flies were subjected to allele-specific PCR (S1 Fig). In addition, *eya^D1^*/*CyO* flies were subjected to PCR with primers eya1F/eyanew2R in exon 5, which do not amplify from the *CyO* chromosome, and no product was generated from the eyaD1 chromosome, consistent with a loss of exon 5. Finally, the genetic background on which the *eya^D1^* allele was generated carries a NotI RFLP in exon 3, as verified by sequence the *eya^D3^* allele that was generated in the same screen; however, PCR and subsequent NotI digestion using *eya^D1^*/*CyO* genomic DNA as a template showed no evidence of restriction activity, implying that amplification only occurred from the *CyO* chromosome that lacks the NotI RFLP. In sum, our allele-specific PCR and RFLP data indicate that the *eya^D1^* allele carries a deletion of the majority of the *eya* regulatory and coding regions.

Several other stocks carrying previously isolated *eya* alleles were not further analyzed due to their lack of eye phenotypes required for our study. In particular, *eya^EY13242^*, an insertion of a *P[EPgy2]* P element just upstream of the *eya-A* promoter [4], showed no eye phenotype as either a homozygote or in combination with *Df(2L)eya*, a large deficiency for the *eya* locus (data not shown). In addition, *eya^137.39^, eya^117.36^,* and *eya^7.42^*, which were isolated based on a gonadogenesis phenotype [5], each results in lethality as homozygotes and in partial lethality as *trans*-heterozygotes with one another, but showed no gross adult eye defects in escaper flies. Furthermore, each fully complements other Class C *eya* alleles *eya^E1^*, *eya^E4^*, and *eya^cliIID^* for viability and eye phenotypes, with the exception of *eya^7.42^*/ *eya^cliIID^*, which produces tight lethality (0/78 homozygotes in inter se cross, p<0.001, χ^2^ test; data not shown).

**References**

1. Bui QT, Zimmerman JE, Liu H, Bonini NM. Molecular analysis of Drosophila eyes absent mutants reveals features of the conserved Eya domain. Genetics. 2000;155(2):709-20. PubMed PMID: 10835393; PubMed Central PMCID: PMC1461105.

2. Bonini NM, Leiserson WM, Benzer S. The eyes absent gene: genetic control of cell survival and differentiation in the developing Drosophila eye. Cell. 1993;72(3):379-95. PubMed PMID: 8431945.

3. Bonini NM, Leiserson WM, Benzer S. Multiple roles of the eyes absent gene in Drosophila. Developmental biology. 1998;196(1):42-57. doi: 10.1006/dbio.1997.8845. PubMed PMID: 9527880.

4. Bellen HJ, Levis RW, Liao G, He Y, Carlson JW, Tsang G, et al. The BDGP gene disruption project: single transposon insertions associated with 40% of Drosophila genes. Genetics. 2004;167(2):761-81. Epub 2004/07/09. doi: 10.1534/genetics.104.026427. PubMed PMID: 15238527; PubMed Central PMCID: PMCPMC1470905.

5. Weyers JJ, Milutinovich AB, Takeda Y, Jemc JC, Van Doren M. A genetic screen for mutations affecting gonad formation in Drosophila reveals a role for the slit/robo pathway. Developmental biology. 2011;353(2):217-28. doi: 10.1016/j.ydbio.2011.02.023. PubMed PMID: 21377458; PubMed Central PMCID: PMCPMC3635084.
