# Supplementary figures and images for: Two modes of transvection at the eyes absent gene of Drosophila demonstrate plasticity in transcriptional regulatory interactions in cis and in trans
Source: PLoS Genet. 2019 May 10;15(5):e1008152. doi: 10.1371/journal.pgen.1008152 (PMC6530868; doi:10.1371/journal.pgen.1008152)

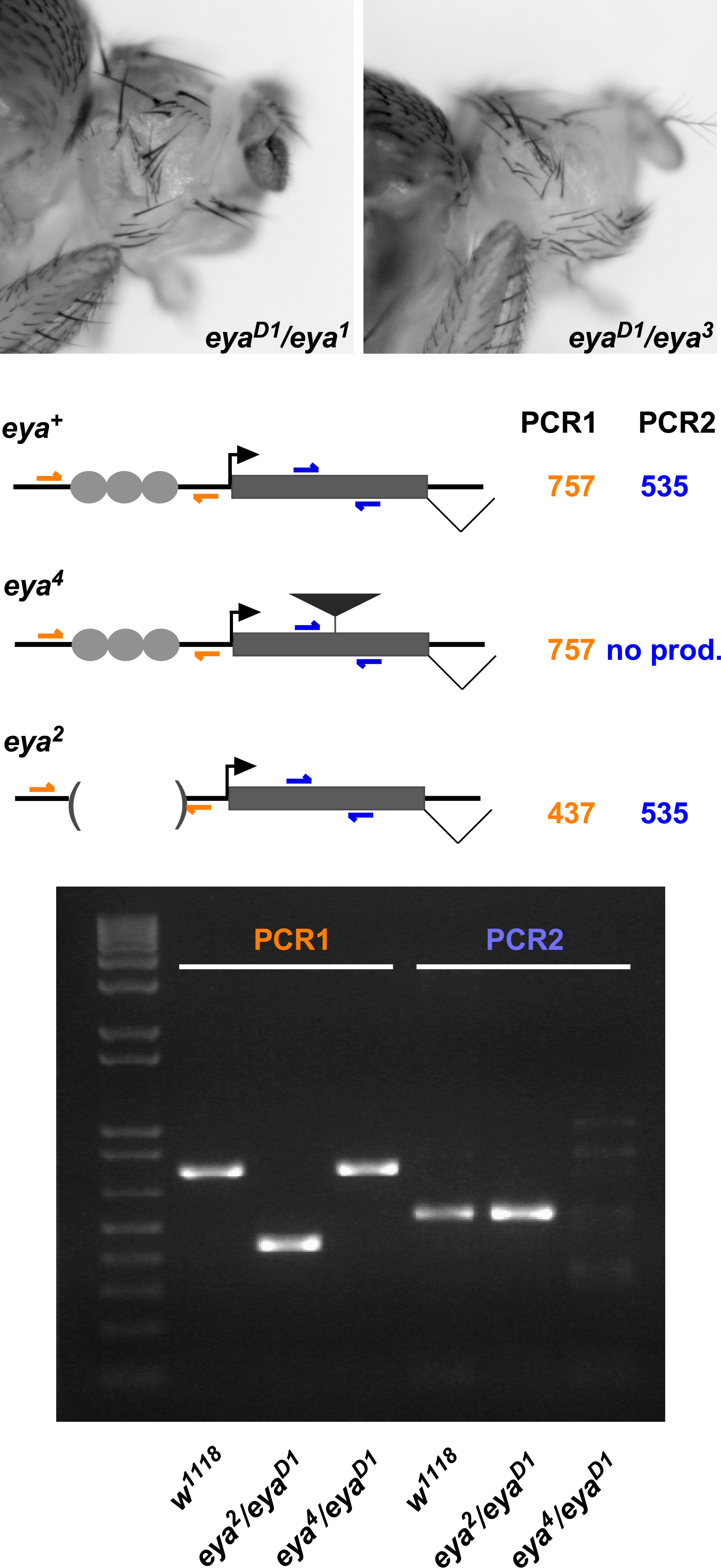

Supplement: S1 Fig — Top, eyaD1 fails to complement the eye phenotypes of Class A and Class B alleles. Below, strategy for characterization of eyaD1 using allele-specific PCR. Primer pairs HopFinder_JTR_F2/HopFinder_JTR_R1 (orange) and eya_P_R1_seq/eya_P_F2_seq (blue) show distinct amplification patterns from eya2 and eya4 chromosomes, respectively. PCR from eyaD1/eya2 and eyaD1/eya2 trans-heterozygotes shows no evidence of amplification from the eyaD1 chromosome. (TIF) [file pgen.1008152.s002.tif]

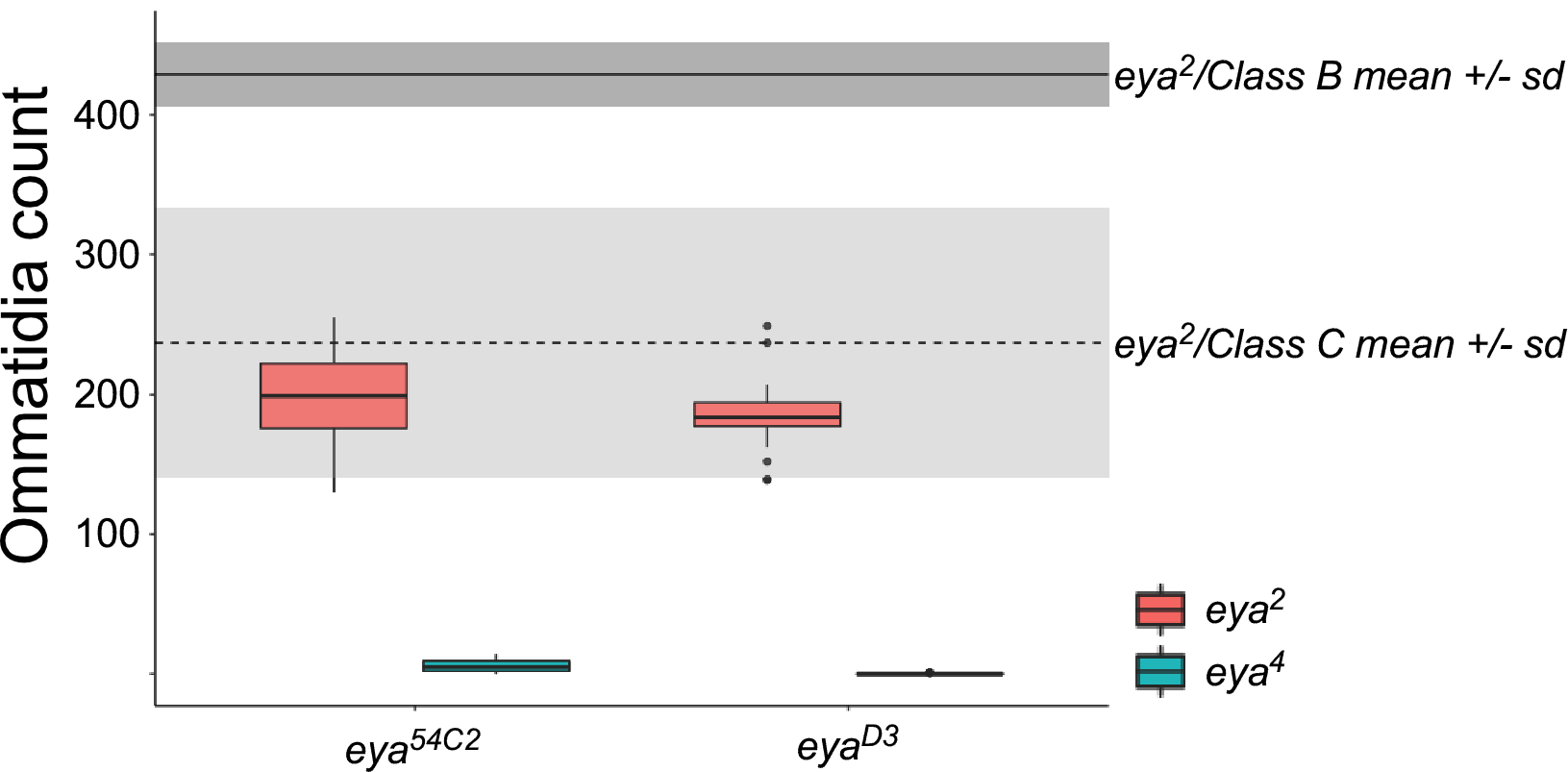

Supplement: S2 Fig — Counts of ommatidia for eya54C2/eya2 and eyaD3/eya2 show complementation consistent with other Class C alleles. Solid line and dark shading represent mean ommatidia counts for eya2 complementation by Class B alleles, dashed line and light shading represent mean ommatidia counts for eya2 complementation by other Class C alleles (see Fig 3). Both alleles completely fail to complement eya4. Data represent n = 20 eyes for each genotype. (TIF) [file pgen.1008152.s003.tif]

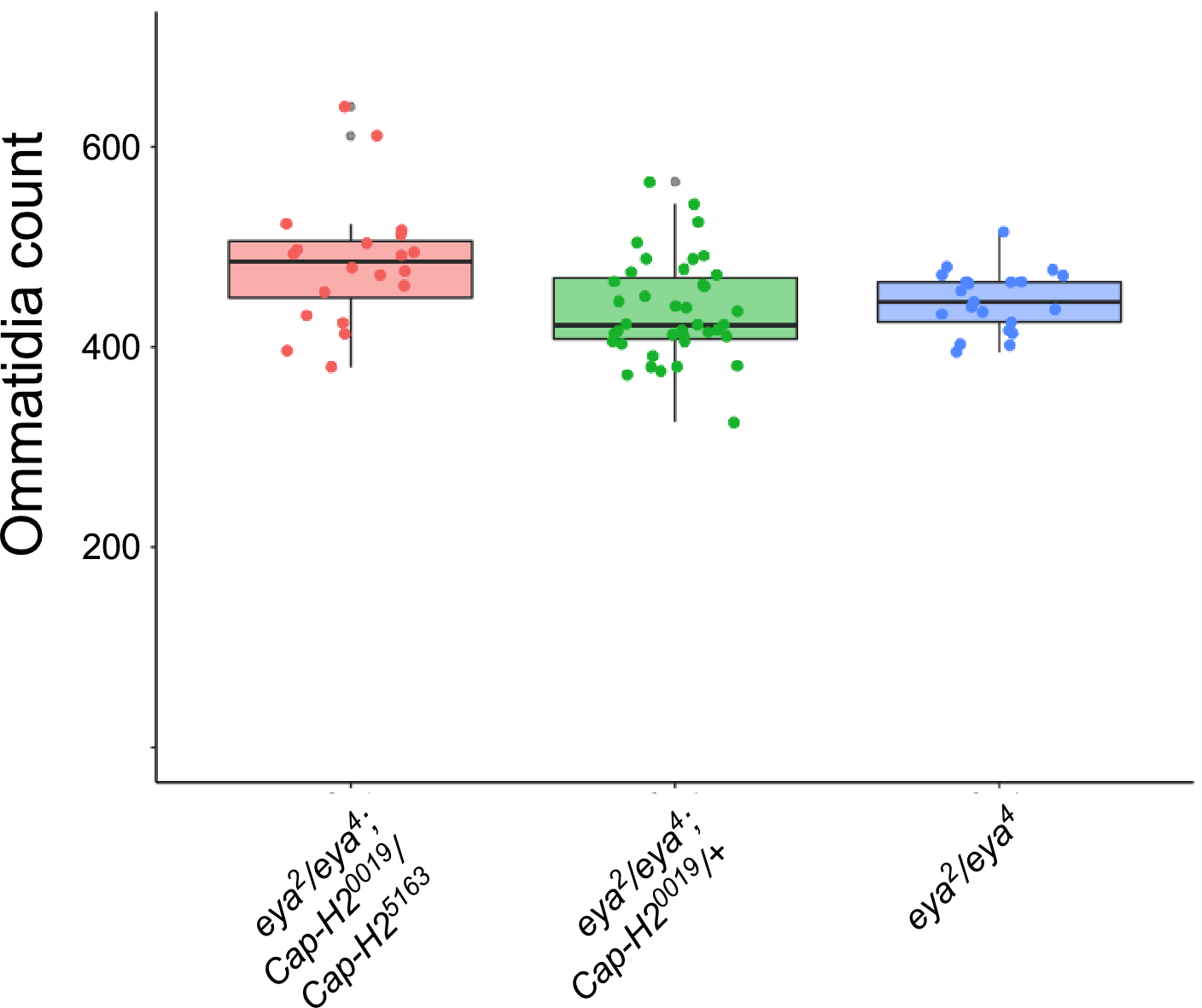

Supplement: S3 Fig — Counts of ommatidia are significantly higher in eya2/eya4; Cap-H20019/Cap-H25163, carrying a strong trans-heterozygous loss of function combination of Cap-H2 mutations, than in eya2/eya4; Cap-H20019/+, which carries one wild-type copy of Cap-H2 (p = 0.003, Mann-Whitney test), or eya2/eya4, where both copies of Cap-H2 are wild type (p = 0.02). (TIF) [file pgen.1008152.s004.tif]
