# Supplementary material for: Two modes of transvection at the eyes absent gene of Drosophila demonstrate plasticity in transcriptional regulatory interactions in cis and in trans
Source: PLoS Genet. 2019 May 10;15(5):e1008152. doi: 10.1371/journal.pgen.1008152 (PMC6530868; doi:10.1371/journal.pgen.1008152)
Supplement: S1 Table — (DOCX) [file pgen.1008152.s005.docx]

**S1 Table. Primers used in this study.**

| **Primer name** | **Primer sequence** | **Use** |
| --- | --- | --- |
| RP49-58F | TACAGGCCCAAGATCGTGAAG | qRT-PCR |
| RP49-175R | GACGCACTCTGTTGTCGATACC | qRT-PCR |
| eyaRT AF1 | AGAATGGTCACCCTAATGCCA | qRT-PCR |
| eyaRT AR1 | TGTGTATCCGTGTGGTCTGTC | qRT-PCR |
| eyaRT F1 | TGCCGCAGGAACAGCGACAG | qRT-PCR |
| eyaRT R1 | CCGTGTGGTCTGTCTTGGGACG | qRT-PCR |
| eyaISA1F | CGCCCGAAGTCGCAGATAAA | RNA *in situ* |
| eyaISA1R | CTTTGGCTCGATCATTTTGTCA | RNA *in situ* |
| eyaISB1F | TGACACGCAGCTTTATGTGAC | RNA *in situ* |
| eyaISB1R | GAAGTTTTGATAGCACGGCACA | RNA *in situ* |
| eyaCRISPR3_F | CTTCGTTGAGAAGGCACTAGGTCAA | sgRNA synthesis |
| eyaCRISPR3_R | AAACTTGACCTAGTGCCTTCTCAA | sgRNA synthesis |
| eya_CRISPR2_G_F | CTTCGAAACTCACTCAAAACTCGCA | sgRNA synthesis |
| eya_CRISPR2_G_R | AAACTGCGAGTTTTGAGTGAGTTTC | sgRNA synthesis |
| eyaCRISPR34check_F | GGAAATGAGCGTACAACTGTCA | PCR to confirm CRISPR editing |
| eyaCRISPR34check_R | GGGCTGGTGTGTTGTTAAGC | PCR to confirm CRISPR editing |
| eyaISA1F | CGCCCGAAGTCGCAGATAAA | RNA *in situ* |
| eyaISA1R | CTTTGGCTCGATCATTTTGTCA | RNA *in situ* |
| eyaISB1F | TGACACGCAGCTTTATGTGAC | RNA *in situ* |
| eyaISB1R | GAAGTTTTGATAGCACGGCACA | RNA *in situ* |
| HopFinder_JTR_R1 | AGTAAGTTAGCAGCTCTGTGATGGCT | characterization/sequencing |
| HopFinder_JTR_F2 | GGGGTTCTTTGGTGCGGGGG | characterization/sequencing |
| Eya_P_R1_seq | GGAACGGCGTCTCCTGGCAC | characterization/sequencing |
| Eya_P_F2_seq | TGCTGGACGGCGATTGGCTG | characterization/sequencing |
| EyaExon2_F | AGAGTTTCAGGCGGTGGAAG | characterization/sequencing |
| EyaExon2_R | AGGATGTTCTCGCCGTTCTG | characterization/sequencing |
| eya seq1F | CCACACTTGACACCACCACCCC | characterization/sequencing |
| eya new2R | AAGTTGTAGGCGCTCAGGTC | characterization/sequencing |
| eya 3F | CCGCAGGCCATGAAACCTGCT | characterization/sequencing |
| eya seq2F | CAACGATCGTAACAATTCTCGCACG | characterization/sequencing |
| eya 2R | GCGGAGATGCGCCAGAAGGG | characterization/sequencing |
| eya seq2R | TCCGGGTCCCAGAAGGGTGC | characterization/sequencing |
| eya1R | CCACACTGCTGCCTCCGCTC | characterization/sequencing |
| eyaseq1R | GAGCCAGTGCCATCGTGCGT | characterization/sequencing |
| eya2F | CCGCCAGCGGCAACAACAAC | characterization/sequencing |
