# Supplementary material for: Two modes of transvection at the eyes absent gene of Drosophila demonstrate plasticity in transcriptional regulatory interactions in cis and in trans
Source: PLoS Genet. 2019 May 10;15(5):e1008152. doi: 10.1371/journal.pgen.1008152 (PMC6530868; doi:10.1371/journal.pgen.1008152)
Supplement: S2 Table — (DOCX) [file pgen.1008152.s006.docx]

**S2 Table. Single Nucleotide Polymorphisms differentiate *eya^E4^* and *eya^54C^* chromosomes.**

| **cDNA position** | **Genbank L08501** | ***eya^E4^*** | ***eya^54C^*** |
| --- | --- | --- | --- |
| 677 | C | G | C |
| 678 | A | G | A |
| 921 | T | T | A |
| 1412 | C | C | T |
| 1541 | G | G | A |
| 1574 | G | G | C |
| 1640 | C | C | A |
| 2282 | T | C | T |

cDNA position is according to Genbank L08501 [2], which describes the *eya-B* transcript (also known as the Type I transcript). Underlining indicates sites where the polymorphism changes the amino acid (678, S to G; 921, S to T relative to the reference sequence). All other changes are silent. *eya^E4^* data was first reported by Bui et al. [1].

1. Bui QT, Zimmerman JE, Liu H, Bonini NM. Molecular analysis of Drosophila eyes absent mutants reveals features of the conserved Eya domain. Genetics. 2000;155(2):709-20. PubMed PMID: 10835393; PubMed Central PMCID: PMC1461105.

2. Bonini NM, Leiserson WM, Benzer S. The eyes absent gene: genetic control of cell survival and differentiation in the developing Drosophila eye. Cell. 1993;72(3):379-95. PubMed PMID: 8431945.
