# Supplementary material for: Two modes of transvection at the eyes absent gene of Drosophila demonstrate plasticity in transcriptional regulatory interactions in cis and in trans
Source: PLoS Genet. 2019 May 10;15(5):e1008152. doi: 10.1371/journal.pgen.1008152 (PMC6530868; doi:10.1371/journal.pgen.1008152)
Supplement: S3 Table — (DOCX) [file pgen.1008152.s007.docx]

**S3 Table. Summary of crosses supporting enhancer action in *trans*.**

|  | ***eya^1^*** | ***eya^2^*** | ***eya^4^*** | ***ETD2.2*** | ***eya^cs^*** |
| --- | --- | --- | --- | --- | --- |
| ***eya^3^*** | 300.3 ± 58.4 (20) | 412.8 ± 56.7 (20) | 0.75 ± 1.44 (20) | 78.4 ± 71.2 (20) | 654.1 ± 110.5 (9) |
| ***eya^4^*** | 326.2 ± 14.8 (20) | 445.2 ± 30.8 (20) | 0.2 ± 0.5 (20) | 188.3 ± 58.7 (20) | 615.4 ± 39.0 (8) |
| ***eya^E1^*** | 45.8 ± 25.5 (20) | 141.25 ± 88.3 (20) | 0.1 ± 0.3 (20) | 0.15 ± 0.67 (20) | *ND* |
| ***eya^E4^*** | 180.3 ± 16.7 (20) | 234.6 ± 43.6 (20) | 3.4 ± 4.1 (20) | 10.7 ± 24.6 (20) | 469.5 ± 43.9 (9) |
| ***eya^cliIID^*** | 190.15 ± 36.1 (20) | 335.55 ± 48.0 (20) | 0.2 ± 0.5 (20) | 0 ± 0 (20) | 477.8 ± 16.0 (5) |
| ***eya^D3^*** | 125.2 ± 77.7 (20) | 186.75 ± 25.6 (20) | 0.1 ± 0.3 (20) | 0.2 ± 0.4 (20) | *ND* |
| ***eya^54C2^*** | *ND* | 198 ± 32.0 (20) | 5.7 ± 4.5 (20) | *ND* | *ND* |
| ***ETD4.3*** | *ND* | *ND* | *ND* | *ND* | 401.6 ± 39.8 (7) |

Data represent mean ommatidia counts plus/minus standard deviation, with the numbers of eyes scored in parentheses.
